# Eulerian–Lagrangian modeling of cough droplets irradiated by ultraviolet–C light in relation to SARS-CoV-2 transmission

**DOI:** 10.1063/5.0039224

**Published:** 2021-03-09

**Authors:** V. D'Alessandro, M. Falone, L. Giammichele, R. Ricci

**Affiliations:** Dipartimento di Ingegneria Industriale e Scienze Matematiche, Università Politecnica delle Marche, Via Brecce Bianche 12, 60131 Ancona (AN), Italy

## Abstract

It is well known that several viruses, as well as SARS-CoV-2, can be transmitted through airborne diffusion of saliva micro-droplets. For this reason, many research groups have devoted their efforts in order to gain new insight into the transport of fluids and particles originated from human respiratory tracts. This paper aims to provide a contribution to the numerical modeling of saliva droplets' diffusion produced by coughing. It is worth noting that droplets' diameters of interest in this work are such that represent typical emission during a cough. Aerosolization effects are neglected since emitted droplets' diameters are greater than 10 *µ*m. In particular, the well-known problem around the safety distance to be held for avoiding virus transmission in the absence of external wind is further investigated. Thus, new indices capable of evaluating the contamination risk are introduced, and the possibility to inactivate virus particles by means of an external ultraviolet-C (UV-C) radiation source is studied. For this purpose, a new model which takes into account biological inactivation deriving from UV-C exposure in an Eulerian–Lagrangian framework is presented.

## I. INTRODUCTION

As largely reported in the open literature, several viruses, as well as SARS-CoV-2, can be transmitted through airborne diffusion of saliva micro-droplets,^1^ too small to be seen with the naked eye. Typical infection mechanisms are the following: (i) direct transfer of large droplets expelled at high momentum to the receiver's conjunctiva, mouth, or nose; (ii) physical contact with droplets deposited on the surface and subsequent absorption to the nasal mucosa of the receiver; and (iii) inhalation by the recipient of respiratory ejected aerosolized droplet nuclei.^2^ For this reason, many countries in the world have imposed variable social distances to be maintained between persons. This restriction has been adopted since the safety distance must be guaranteed in order to allow the most elevated number of droplets to fall down and reach the floor after their emission from a mouth or nose. Hence, it is straightforward to understand that the correct and rigorous study of saliva droplet dynamics, involving all the relevant biological and physical phenomena, is the key ingredient to determine the guidelines on social distancing, face mask wearing, and the implementation of new practices in daily social life. It is also worth noting that the physical phenomena involved in the droplet transmission process are very complex. Indeed, after their emission, micro-droplets travel as a result of their inertia and their aerodynamic interaction with air. Moreover, the mass of the droplet can vary due to evaporation, which is strictly connected to air temperature and relative humidity.

Since the SARS epidemic, which started at the end of 2002, several studies about airborne droplet transmission have been published in medical and non-medical journals. As already introduced, both computational and experimental models have been employed by investigators with particular emphasis on indoor conditions.^3^ Some research groups have carried on chamber experiments. However, an essential disadvantage of this kind of approach is that traditional measurements are too discrete, i.e., only a few points can be investigated at the same time. On the contrary, complex laser-based techniques, such as Particle Image Velocimetry (PIV), allow measuring 2D or even 3D velocity fields. Numerical modeling can be considered a valid tool to study the research topic treated in this paper. Flow fields and droplet dynamics can be computed with a very high temporal resolution, far less than the scale of the human breathing activities. Moreover, computer simulations have more considerable flexibility than experimental investigations. However, an important issue for Computational Fluid-Dynamics (CFD) simulations is the obtained accuracy, which is influenced by geometrical simplifications as well as the failure of adopted models.^4^ After the unprecedented COVID-19 pandemic, several research groups have devoted their efforts in order to gain new insight into the transport of fluids and particles emanating from human respiratory tracts. Both experimental and numerical contributions have appeared in the literature. Wang *et al.*^5^ used the PIV technique to measure the flow field developing near the mouth and particle shadow velocimetry (PSV) to track the trajectories of large droplets. It is important to note that also many papers regarding flow visualization were published. Thus, in this context, Verma *et al.*^6,7^ produced flow visualizations in order to evaluate the effectiveness of protection masks. Similarly, Staymates^8^ performed Schilieren visualizations of flow deriving from face masks, but he used a volunteer and not a mannequin as mentioned above. Arumuru *et al.*^9^ carried out visualizations of sneezing in relation to mask and shield efficacy.

Concerning the numerical approaches, different modeling strategies have been adopted. They can be divided into two main classes: (i) dilute air–saliva mixture and (ii) Eulerian–Lagrangian modeling. The first class includes the effect of saliva dispersion in air by coupling governing equations with a further convection–diffusion equation. The second class of models is based on an Eulerian approach for the atmospheric air, while the Lagrangian reference frame is used for dispersed droplets generated by breathing. Vuorinen *et al.*^10^ discussed the physical processes related to the aerosolization of the exhaled droplets by means of Large Eddy Simulation (LES) and a convection–diffusion equation for saliva concentration. A similar approach was used by Khosronejad *et al.*^11^ with a particular focus on face masks. As a regard of Eulerian–Lagrangian modeling, Pendar and Pascoa^12^ presented a model for coughing and sneezing activities based on a compressible LES approach for the Eulerian phase. Differently, Dbouk and Drikakis^13–15^ relied on the Reynolds-Averaged Navier–Stokes (RANS) equations for the simulation of human cough; the impact of face masks and weather conditions on the droplet evaporation phenomenon were studied. Also, Busco *et al.*^16^ adopted RANS equations to model the carrier fluid under sneezing and asymptomatic conditions, including biomechanics effects. In Abuhegazy *et al.*,^17^ a RANS-based Eulerian–Lagrangian model of bio-aerosol transport in a classroom, with relevance to COVID-19, is discussed. Regarding the impact of human physiology factors driving droplet dispersion, Fontes *et al.*^18^ proposed a model relying on detached eddy simulation. They present an extensive study about the impact of several parameters on saliva droplet diffusion during sneezing. Finally, Li *et al.*^19^ presented a model oriented on the assessment of droplet evaporation and transport in a tropical outdoor environment.

In this paper, a new computational model, relying on the well-established OpenFOAM library,^20^ for the evaluation of saliva droplets' dynamics during coughing is developed. One of the main aims of this work is to provide CFD practitioners with several crucial data about case settings. The second aim is to introduce two new indices in order to evaluate contamination risk. Finally, a focus on the possibility to reduce SARS-CoV-2 transmission potential by means of ultraviolet-C (UV-C) radiation is shown. This topic was already investigated by Buchan *et al.*^21^ In the cited paper, the UV-C effect was assessed considering saliva droplets in a dilute solution with air. The present work aims to introduce an innovative approach, capable of including the biological inactivation related to UV-C radiation, in an Eulerian–Lagrangian framework.

This paper is organized as follows: the governing equations are reported in Sec. II, while the numerical discretization techniques are discussed in Sec. III. Numerical results are shown in Sec. IV. Finally, Sec. V contains the conclusions.

## II. GOVERNING EQUATIONS

Numerical simulations are developed using an Eulerian–Lagrangian framework described in the following.

### A. Eulerian phase

For the Eulerian phase, compressible RANS equations are used,

$$\begin{array}{c} \frac{\partial \bar{\rho }}{\partial t}+\frac{\partial }{\partial x_{j}}\left(\bar{\rho }{\tilde{u}}_{j}\right)=s_{m},\\ \frac{\partial }{\partial t}\left(\bar{\rho }{\tilde{u}}_{i}\right)+\frac{\partial }{\partial x_{j}}\left(\bar{\rho }{\tilde{u}}_{i}{\tilde{u}}_{j}\right)=-\frac{\partial \bar{p}}{\partial x_{i}}+\frac{\partial {\hat{\tau }}_{ij}}{\partial x_{j}}+\bar{\rho }g\delta_{i3}+s_{m,i},\\ \frac{\partial }{\partial t}\left(\bar{\rho }\tilde{E}\right)+\frac{\partial }{\partial x_{j}}\left(\bar{\rho }{\tilde{u}}_{j}\tilde{H}\right)=-\frac{\partial q_{j}}{\partial x_{j}}+\frac{\partial }{\partial x_{j}}\left({\tilde{u}}_{i}{\hat{\tau }}_{ij}\right)+s_{e},\\ \frac{\partial }{\partial t}\left(\bar{\rho }{\tilde{Y}}_{k}\right)+\frac{\partial }{\partial x_{j}}\left(\bar{\rho }{\tilde{u}}_{j}{\tilde{Y}}_{k}\right)=-\frac{\partial m_{k,j}}{\partial x_{j}}+s_{Y_{k}}, \end{array}$$ (1)

where $\bar{\rho }$, ${\tilde{u}}_{i}$, $\bar{p}$, $\tilde{T}$, and $\tilde{Y_{k}}$ denote density, velocity component in the *x*_*i*_ direction, pressure, temperature, and chemical specie *k* mass fraction, respectively. $\tilde{E}$ and $\tilde{H}$ are, respectively, the total internal energy and enthalpy. Note that the overbar and the tilde are filtering operators which are introduced for unweighted and density-weighted averages, respectively.

The unclosed terms reported in Eq. (1) are handled as follows:

$$\begin{array}{c} q_{j}=-c_{p}\left(\frac{\mu }{\mathrm{Pr}}+\frac{\mu_{t}}{{Pr}_{\text{t}}}\right)\frac{\partial \tilde{T}}{\partial x_{j}},\\ m_{k,j}=-\left(\frac{\mu }{{Sc}_{\text{k}}}+\frac{\mu_{t}}{{Sc}_{k,\;t}}\right)\frac{\partial {\tilde{Y}}_{k}}{\partial x_{j}}. \end{array}$$ (2)

In Eq. (2), the symbol *c*_*p*_ represents the specific heat at constant pressure and *μ* is the viscosity. Pr and Sc_k_ are molecular Prandtl and Schmidt numbers, respectively; the lower-script *t* indicates the turbulent version of the previous dimensionless groups. The stress tensor ${\hat{\tau }}_{ij}$ is evaluated as follows:

$${\hat{\tau }}_{ij}=2\mu \left({\tilde{S}}_{ij}-\frac{1}{3}\frac{\partial {\tilde{u}}_{k}}{\partial x_{k}}\delta_{ij}\right)+\tau_{ij},$$ (3)

where

$$\tau_{ij}=2\mu_{t}\left({\tilde{S}}_{ij}-\frac{1}{3}\frac{\partial {\tilde{u}}_{k}}{\partial x_{k}}\delta_{ij}\right)-\frac{2}{3}\bar{\rho }\bar{k}\delta_{ij},$$ (4)

where $\bar{k}$ is the average turbulent kinetic energy and ${\tilde{S}}_{ij}$ is the strain-rate tensor. Turbulence modeling is performed using standard Shear Stress Transport (SST) *k*–*ω*, developed by Menter,^22^ not described here for compactness. The polynomial equation of state was adopted, and polynomial correlations were used for thermophysical properties.

The source terms *s*_*m*_, *s*_*m*,*i*_, *s*_*e*_, and $s_{Y_{k}}$ correspond to coupling between Lagrangian and Eulerian phases with respect to mass, momentum, energy, and species, respectively. The particle-source-in-cell (PSI–cell) method^23^ for source terms manipulation is adopted.

### B. Lagrangian phase

Saliva droplets are tracked using a Lagrangian frame throughout the computational domain. It is crucial to put in evidence that, within OpenFOAM Lagrangian libraries, for efficiency reasons, the concept of computational parcel is adopted. The droplets are organized in groups, and each parcel represents the center of mass of a small cloud of droplets having the same properties. Assuming non-collisional spherical parcels, position and velocity are the results of the trajectory and momentum equations,

$$\begin{array}{c} \frac{dx_{P,i}}{dt}=u_{P,i},\\ m_{P,i}\frac{du_{P,i}}{dt}=F_{P,i}^{G}+F_{P,i}^{D}, \end{array}$$ (5)

with parcel velocity **u**_*P*,*i*_, mass *m*_*P*,*i*_, and position **x**_*P*,*i*_. The forces acting on the generic *i*th parcel in Eq. (5) are identifiable with two contributions: gravity force ( $F_{P,i}^{G}$) and aerodynamic drag force ( $F_{P,i}^{D}$). Gravity force takes also into account buoyancy in the following way:

$$F_{P,i}^{G}=m_{P,i}g\left(1-\frac{\bar{\rho }}{\rho_{P}}\right),$$ (6)

where *ρ*_*P*_ is the density of the generic element of the discrete phase. The aerodynamic drag force, $F_{P,i}^{D}$, is

$$F_{P,i}^{D}=\bar{\rho }C_{D}\frac{\pi D_{P,i}^{2}}{8}\left(\tilde{u}-u_{P,i}\right)\left|\tilde{u}-u_{P,i}\right|,$$ (7)

where the drag coefficient, *C*_*D*_, is evaluated from a correlation based on Putnam^24^ paper where *D*_*P*,*i*_ is the particles' diameter. Additional forces including the following components, pressure, virtual mass, Basset, and Brownian, are not included as done by other authors.^16,17,19^ As a matter of fact, the particles considered in the present work are sufficiently small to neglect pressure and virtual mass forces and sufficiently large to neglect Brownian force.^17,25,26^ This evidence held true also in the preliminary computations carried out in this research.

The mass conservation equation reads

$$\frac{dm_{P,i}}{dt}=-{\dot{m}}_{P,i}^{ev},$$ (8)

where the evaporation term, ${\dot{m}}_{P,i}^{ev}$, is governed by the diffusive flux of vapor and the mass transfer coefficient is obtained from the well-established Ranz–Marshall correlation.^27^

Finally, the parcel temperature, *T*_*P*,*i*_, is obtained through the analytic solution of the energy equation,

$$m_{P,i}c_{p,i}\frac{dT_{P,i}}{dt}=hA_{P,i}\left(T_{P,i}-\tilde{T}\right)+{\dot{Q}}_{ev}.$$ (9)

In Eq. (9), the convective heat transfer coefficient, *h*, is obtained from the Ranz–Marshall correlation^27^ for Nu number; ${\dot{Q}}_{ev}$ is the term including the heat transfer between continuous and discrete phases due to droplet evaporation.

The Rosin–Rammler distribution^28^ is used for representing initial parcels' diameter,

$$f=\frac{n}{{\bar{D}}_{P}}{\left(\frac{D_{P,i}}{{\bar{D}}_{P}}\right)}^{n-1}\;\exp\left[-{\left(\frac{D_{P,i}}{{\bar{D}}_{P}}\right)}^{n}\right].$$ (10)

In Eq. (10), we fix *n* = 8 and the average parcels' diameter, ${\bar{D}}_{p}$, equal to 80 *µ*m as in Dbouk and Drikakis^14^ who performed a fit of Xie *et al.*^29^ experimental data regarding human cough. The minimum diameter of the injected parcels is 10 *µ*m, while the maximum one is 280 *µ*m.

### C. UV-C inactivation modeling

The present work shows a new model able to take into account the presence of virus/bacterial particles in a saliva droplets' cloud and evaluate their biological inactivation produced by an external UV-C field.

This is a complex multi-physics problem, and it was addressed in the available literature by solving a transport equation for virus concentration.^21,30,31^ Similar approaches can be considered appropriate for handling dilute solutions, but they are not suitable to investigate the interaction of UV-C light with a cloud of saliva droplets produced during cough or sneeze.

In the approach described here, the number of active particles in each parcel, *N*_*a*,*i*_, is estimated starting from the number of particles grouped inside the parcel itself, *N*_*p*,*i*_, as follows:

$$N_{a,i}=N_{p,i}-I_{a,i},$$ (11)

where

$$\begin{array}{c} I_{a,i}\left(t^{\left(k\right)}\right)=\overset{N_{ts}}{\underset{k=1}{\sum }}N_{a,i}\left(t^{\left(k-1\right)}\right)F_{a,i}\\ F_{a,i}=1-e^{-ZE_{p}\left(x_{P,i},t^{\left(k\right)}\right)\Delta t}. \end{array}$$ (12)

In Eq. (12), the term *I*_*a*,*i*_ is the number of particles inactivated by UV-C radiation in the parcel focusing on the point **x**_*P*,*i*_; the integer parameter, *N*_*ts*_, represents the current time–step index. It is important to remark that the inactivation coefficient, *F*_*a*,*i*_, is derived from the first-order Chick–Watson kinetics,

$$\frac{N\left(t\right)}{N_{0}}=e^{-ZE_{p}t}.$$ (13)

In Eq. (13), $N\left(t\right)$ and *N*_0_ represent the number of active particles at the generic time instant *t* and *t* = 0, respectively. Differently, *Z* is a susceptibility constant for the microorganism and *E*_*p*_ is the mean irradiance of the UV-C field. In this research work, we fix *Z* = 8.5281 · 10^−2^ m^2^/J which is the average experimental value^32^ obtained for UV-C light (*λ* = 254 nm) irradiating SARS-CoV-2.

With regard to *E*_*p*_, its estimation is achieved by means of the thermal radiation view factor method.^33^ This technique was chosen for its capability in well describing the intensity field due to cylindrical UV lamps.^34^ The fraction of the total radiation intensity emitted, which is collected by a parcel perpendicular to the lamp axis and located in correspondence of its edge, is given by

$$\begin{array}{l} F=\frac{L_{l}}{\pi H_{l}}\left[\frac{1}{L_{l}}\arctan\left(\frac{L_{l}}{\sqrt{H_{l}^{2}-1}}\right)-\arctan\left(M\right)\right.\\ \left.+\frac{X-2H_{l}}{\sqrt{XY}}\arctan\left(M\sqrt{\frac{X}{Y}}\right)\right]. \end{array}$$ (14)

The parameters in Eq. (14) are based on the length of the lamp axis, *l*, its radius, *r*, and the distance from the lamp, *d*. They are calculated as follows:

$$\begin{array}{c} H_{l}=\frac{d}{r},\;L_{l}=\frac{l}{r},\;X=\left(1+H_{l}^{2}\right)+L_{l}^{2},\\ Y=\left(1-H_{l}^{2}\right)+L_{l}^{2},\;M=\sqrt{\frac{H_{l}-1}{H_{l}+1}}. \end{array}$$ (15)

The total view factor, *F*_*tot*_, is actually adopted to evaluate the UV-C irradiance field reaching a parcel. For points located between lamp edges, *F*_*tot*_ is given by the superposition of two different contributions,

$$F_{tot}=F\left(l_{1}\right)+F\left(l_{2}\right).$$ (16)

It is worth noting that *l*_1_ and *l*_2_ are segments deriving from lamp splitting in correspondence of the parcel position. Hence, *F*(*l*_1_) and *F*(*l*_2_) are the results of the application of Eq. (14) to the lamp portions. Differently, for parcels located beyond or before the lamp ends, a “ghost” length, *l*_*g*_, is considered. This length is the axial distance between the parcel and lamp edge. In this case, the total view factor is calculated by subtracting the ghost portion contribution as follows:

$$F_{tot}=F\left(l+l_{g}\right)-F\left(l_{g}\right).$$ (17)

Finally, *F*_*tot*_ is used to evaluate the UV-C field intensity on each parcel present in the domain as a function of its distance from the lamp axis,

$$E_{p}=\frac{W_{l}}{2\pi rl}F_{tot}.$$ (18)

Here, *W*_*l*_ is the total power of the lamp.

## III. NUMERICAL APPROXIMATION

The governing equation solution relies on the OpenFOAM library. Thus, the unstructured, colocated, cell-centered finite volume method was adopted for space discretization. An implicit, three level, second-order scheme was used for the time integration together with the dynamic adjustable time stepping technique for guaranteeing a local Courant (Co) number less than a user-defined value (Co_max_). The interpolation of convective fluxes is treated by the linear upwind scheme, whereas diffusive terms are discretized by a standard second-order central scheme. Moreover, pressure–velocity coupling is handled through the Pressure-Implicit with Splitting Operators (PISO) procedure.^35^ For the linear solvers, a preconditioned conjugate gradient (PCG) method with a diagonal incomplete-Cholesky preconditioner was used to solve the pressure equation. A preconditioned bi-conjugate gradient (PBiCG) method with the Diagonal Incomplete Lower Upper (DILU) preconditioner was adopted instead for the remaining equations. In particular, a local accuracy of 10^−7^ was established for the pressure, whereas other linear systems were considered as converged when the residuals reached the machine precision.

### A. Computational grids

**FIG. 1.**
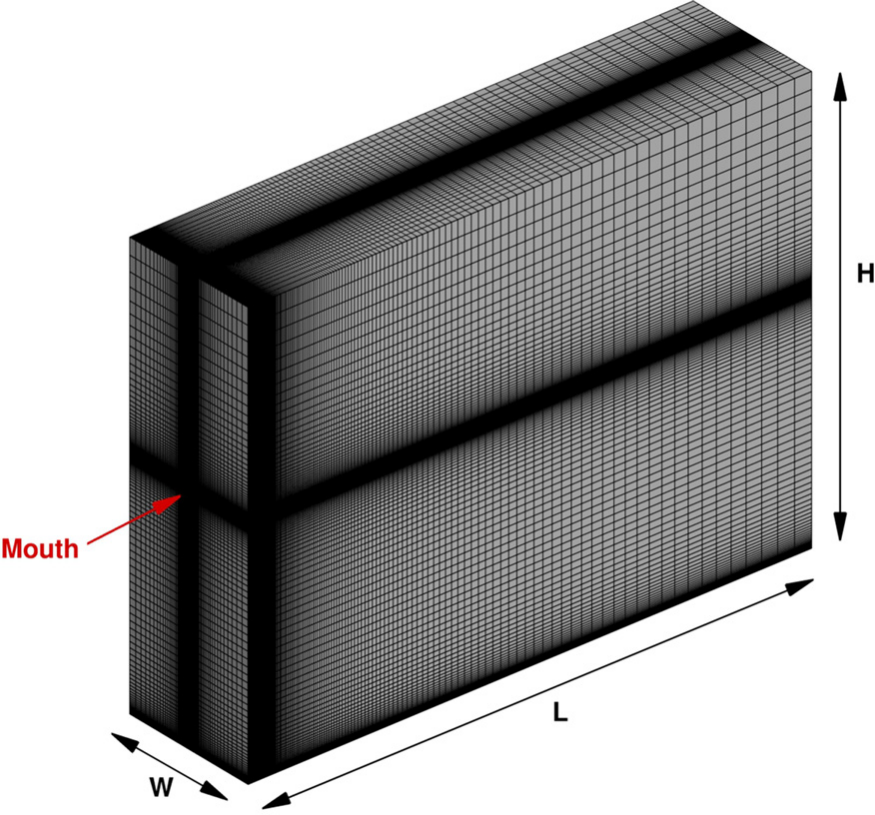
Computational grid representation.

**FIG. 2.**
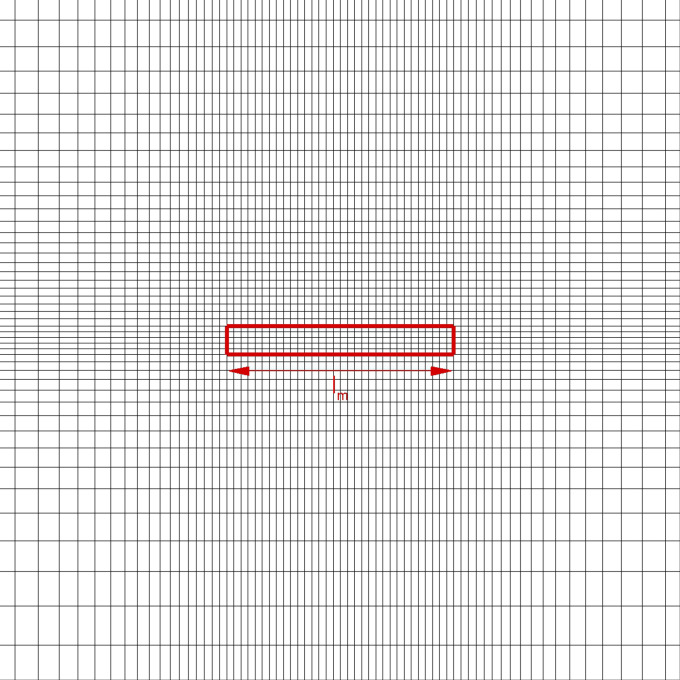
Fully structured mesh, mouth print refinement.

In the present work, a 3D computational domain was considered, as shown in Fig. 1. It consists of an air volume starting from the mouth print of a standing coughing person. A length *L* = 4 m, a width *W* = 1 m, and a height *H* = 3 m were adopted, in accordance with Dbouk and Drikakis.^13^ The mouth print is approximated as rectangular, having a length of *l*_*m*_ = 0.04 m and a total area of *A*_*m*_ = 2 · 10^−4^ m^2^. The reference frame origin, $O=\left(0,0,0\right)$, is fixed in the same plane where the mouth print is placed, see Figs. 1 and 2. In particular, *O* is in the middle of the previous face in correspondence with the intersection of the domain bottom side. *X*-axis is aligned with the droplet propagation direction; *y*-axis represents the transverse direction, while *z*-axis is the vertical direction.

The mouth print center, *P*_*m*_, was placed in the same position selected by Dbouk and Drikakis:^13^ *P*_*m*_ = (0, 0, 1.63).

**TABLE I.** Grid point distribution.

Grid	L points	W points	H points	Total
S1	170	112	150	2.856 · 10^6^
S2	221	136	194	5.830 · 10^6^
S3	270	158	231	9.854 · 10^6^

Fully structured meshes were built in order to discretize the domain. A suite of three different grids named *S*1, *S*2, and *S*3 was generated using, for all the cases, 160 cells for mouth discretization. Besides, mesh element size grading was applied so as to achieve a proper discretization in the droplets' emission and transport areas (Fig. 2). The height of the first cell next to the ground, *z*_*c*_, was set at 10^−3^ m. Other grid details are collected in Table I.

### B. Initial and boundary conditions

A stepped velocity inlet at the mouth boundary, with injection of parcels, was applied to mimic the human cough over 0.12 s. The velocity inlet value was deduced on the base of measurements carried on by Scharfman *et al.*,^36^ and it is equal to 8.5 m/s in the streamwise direction both for the carrier fluid and injected parcels. In the same boundary, turbulence intensity, Tu, is fixed at 15% and the mixing length is equal to 7 × 10^−3^. Furthermore, the initial total mass of saliva droplets laden into the domain for a single cough is 7.7 mg, according to the experimental measurements performed by Xie *et al.*^29^ and CFD simulations of Dbouk and Drikakis.^13^ Saliva is, in general, a complex fluid but, following Van Der Reijden *et al.*,^37^ it could be approximated as water. For this reason, the impact of the UV-C field on the parcels' temperature is neglected since UV-C water absorptivity is extremely low. The remaining part of the *y*–*z* plane at *x* = 0 m is such that all the variables have a null gradient through it. The bottom side of the domain, i.e., the ground, is modeled as a standard wall. The symmetry condition is imposed on lateral boundaries: *y* = ±0.5 m. On the other hand, the zero gradient condition is set for all variables at the domain top with the exception of the pressure. In this case, the pressure is reduced to its hydrostatic level. The *y*–*z* plane at *x* = 4 m is managed as a physical outflow. However, the pressure is imposed to decrease linearly, starting from the atmospheric pressure level at *z* = 0.

The initial temperature of the carrier fluid is 20 °C with relative humidity fixed at 50%. The ground is at 25 °C, while the air and droplets ejected by human mouth are at 34 °C. The initial mass fraction composition of the Eulerian phase is as follows: 0.991 dry air and 0.009 water vapor as in Dbouk and Drikakis.^13^ The hypothesis of injection of saturated moist air from the mouth is also taken into account in the present work.

The estimated maximum Weber number is smaller than the critical one,^38^ and this is the reason why any secondary breakup model is introduced in the following computations.

**FIG. 3.**
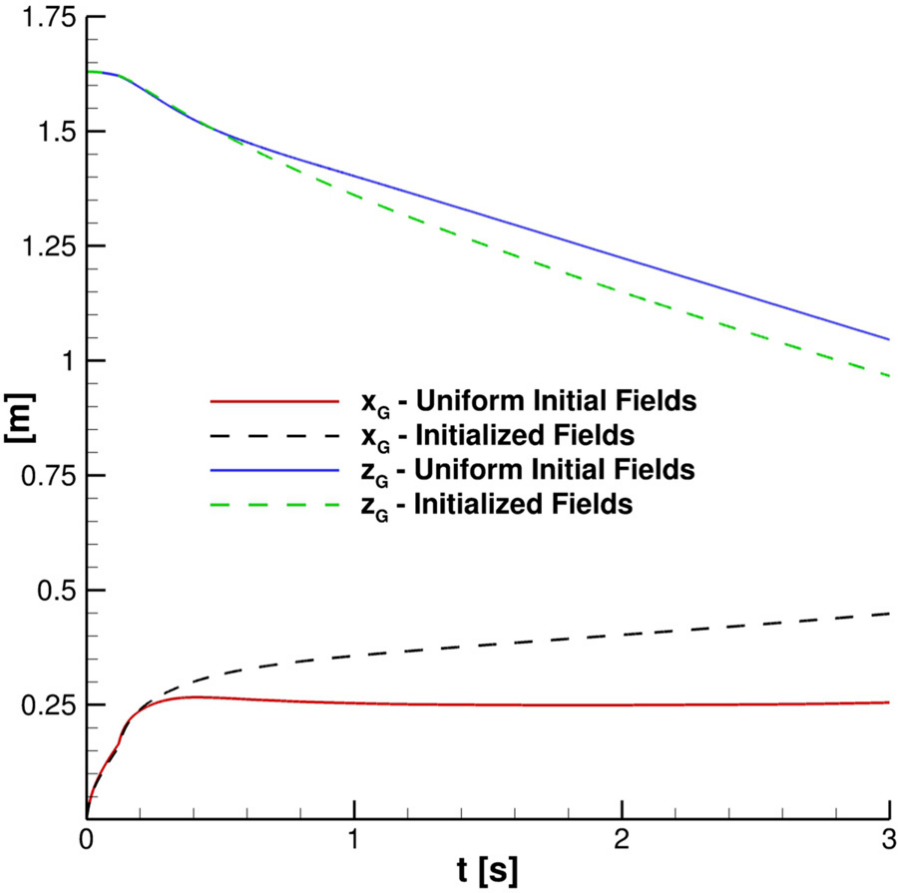
Effect of the initial conditions on the particles' cloud evolution. S2 grid, Co_max_ = 0.2.

It is very important to put in evidence that previous initialization is used to generate more realistic initial fields for full runs, consistent with atmospheric conditions. In this precursor simulation, no mass is introduced from the mouth print boundary which is treated like the remaining part of the *x* = 0 plane. The preliminary simulation stage is considered completed when a physical time of 15 s is reached and, only after, parcel injection starts. Subsequently, in this precursor, turbulent variables are initialized and the hydrostatic pressure field is obtained. It is worth emphasizing that cloud evolution is strongly influenced, as represented in Fig. 3. Indeed, the cloud center of mass position (defined in Sec. IV) in time completely changes when non-uniform initial fields are employed. Note that uniform initial fields produce an underestimation of the cloud axial penetration.

## IV. RESULTS

The present section shows the obtained numerical results referred to the saliva droplets produced during coughing as a cloud. Several cloud characteristics are calculated and considered in order to investigate its diffusion and interaction with artificial UV-C light, i.e., (i) the cloud center of mass, (ii) streamwise liquid penetration length, (iii) fraction of particles present in a reference volume, (iv) active fraction in a reference volume, and (v) particle weighted average diameter. The cloud center of mass is computed as follows:

$$G=\frac{\overset{\hat{N_{p}}\left(\Omega_{0}\right)}{\underset{i=1}{\sum }}m_{P,i}x_{P,i}}{\overset{\hat{N_{p}}\left(\Omega_{0}\right)}{\underset{i=1}{\sum }}m_{P,i}},$$ (19)

where $\hat{N_{p}}\left(\Omega_{0}\right)$ is the number of parcels laden in the overall domain, Ω_0_, in a given time-instant. In the following lines, $G=\left(x_{G},y_{G},z_{G}\right)$ is considered as the center of mass components.

Streamwise liquid penetration length, LPL_x_, is defined as the maximum distance traveled along the *x*-axis by a parcel conserving at least 95% of its initial mass. It is interesting to put in evidence that for this parameter, publicly available OpenFOAM functions are not used. Thus, an inline function leaning on the parcel mass stored at the domain immission is developed. Two different indices for describing the saliva droplets' population/activation are introduced. The first index is the ratio between the number of particles present in a reference volume, Ω_*i*_, and the total number of particles in Ω_0_ in a given time instant

$$\Phi_{\Omega_{i}}=\frac{\overset{\hat{N_{p}}\left(\Omega_{i}\right)}{\underset{k=1}{\sum }}N_{p,k}}{\overset{\hat{N_{p}}\left(\Omega_{0}\right)}{\underset{k=1}{\sum }}N_{p,k}}.$$ (20)

A second reference index is expressed in the following equation:

$$\Phi_{A,ij}=\frac{\overset{\hat{N_{p}}\left(\Omega_{j}\right)}{\underset{k=1}{\sum }}N_{a,k}}{\overset{\hat{N_{p}}\left(\Omega_{i}\right)}{\underset{k=1}{\sum }}N_{p,k}}.$$ (21)

Here, Φ_*A*,*ij*_ is the ratio of active particles in Ω_*j*_ and the number of particles hosted in Ω_*i*_. The aim of the Φ_*A*,*ij*_ index is to provide a quantitative analysis of the impact of UV-C related biological inactivation.

It is essential to remark that the data presented in this paper are focused on parallelepipedal reference volumes having the following features:

$$\Omega_{i}=\left[0,\alpha_{i}\right]\times \left[-0.5,0.5\right]\times \left[1.3,1.8\right].$$ (22)

The parameter *α*_*i*_, appearing in Eq. (22), spans the following values: 0.5 m, 1.0 m, 1.2 m, and 1.5 m, which are selected in order to investigate a proper safety distance to be held in context of SARS-CoV-2 transmission containment. The transverse direction range is considered in order to completely cover the domain. The *z*-axis interval is defined for acting on a sufficiently wide range of possible virus receivers' heights.

Finally, the impact of droplets' evaporation is assessed using the particle weighted average diameter, *D*_10_, as follows:

$$D_{10}=\frac{\overset{\hat{N_{p}}\left(\Omega_{0}\right)}{\underset{i=1}{\sum }}N_{P,i}D_{P,i}}{\overset{\hat{N_{p}}\left(\Omega_{0}\right)}{\underset{i=1}{\sum }}N_{P,i}}.$$ (23)

All the computations were performed on the high-performance computing (HPC)-system CRESCO6 hosted by ENEA at Portici (Italy). CRESCO6 comprises 434 nodes with two Intel Xeon Platinum 8160 24-core processors of the Skylake (SKL) generation operating at 2.1 GHz for each node. There is 192 GB RAM available in the standard nodes. The codes were built using Intel compilers and the MPI library version developed by Intel.

### A. Grid convergence study

**FIG. 4.**
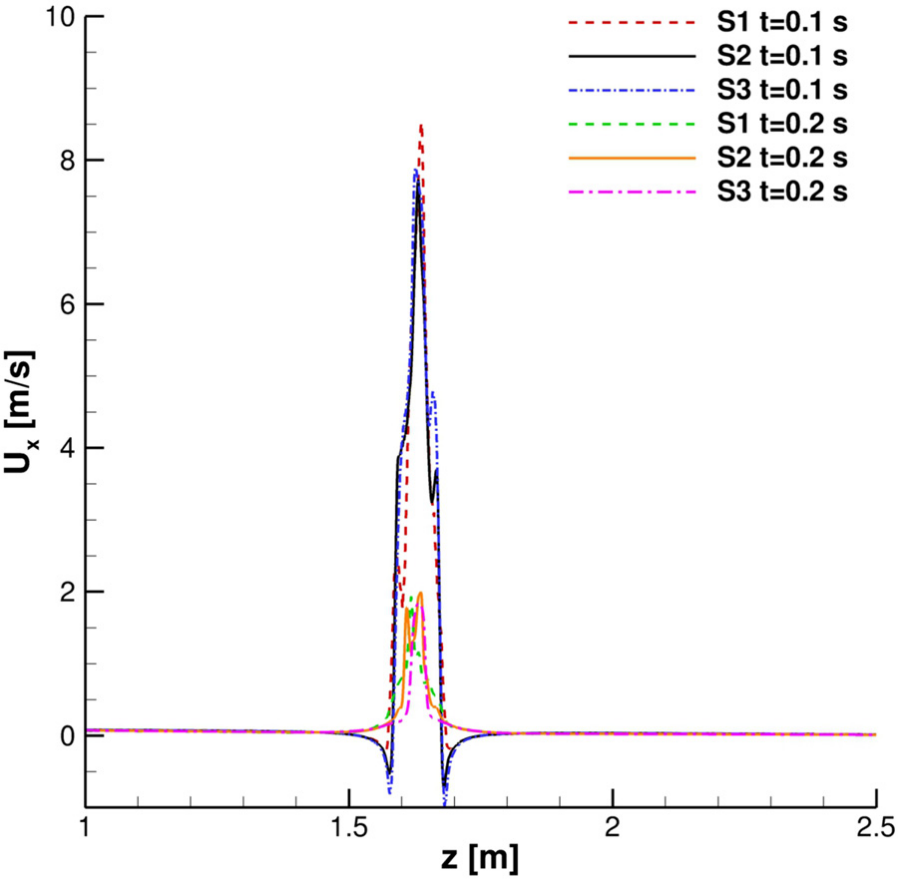
Streamwise velocity profiles. *x* = 0.15 m, *y* = 0 m, and *t* = 0.1 s and 0.2 s.

**FIG. 5.**
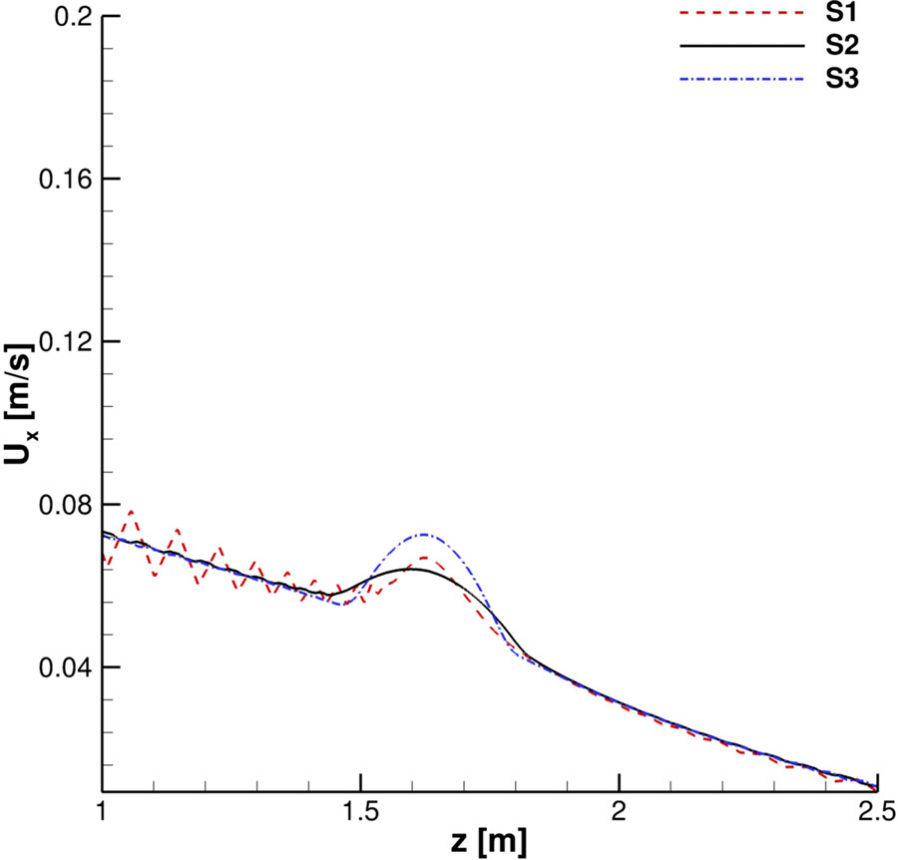
Streamwise velocity profiles. *x* = 0.15 m, *y* = 0 m, and *t* = 1 s.

**FIG. 6.**
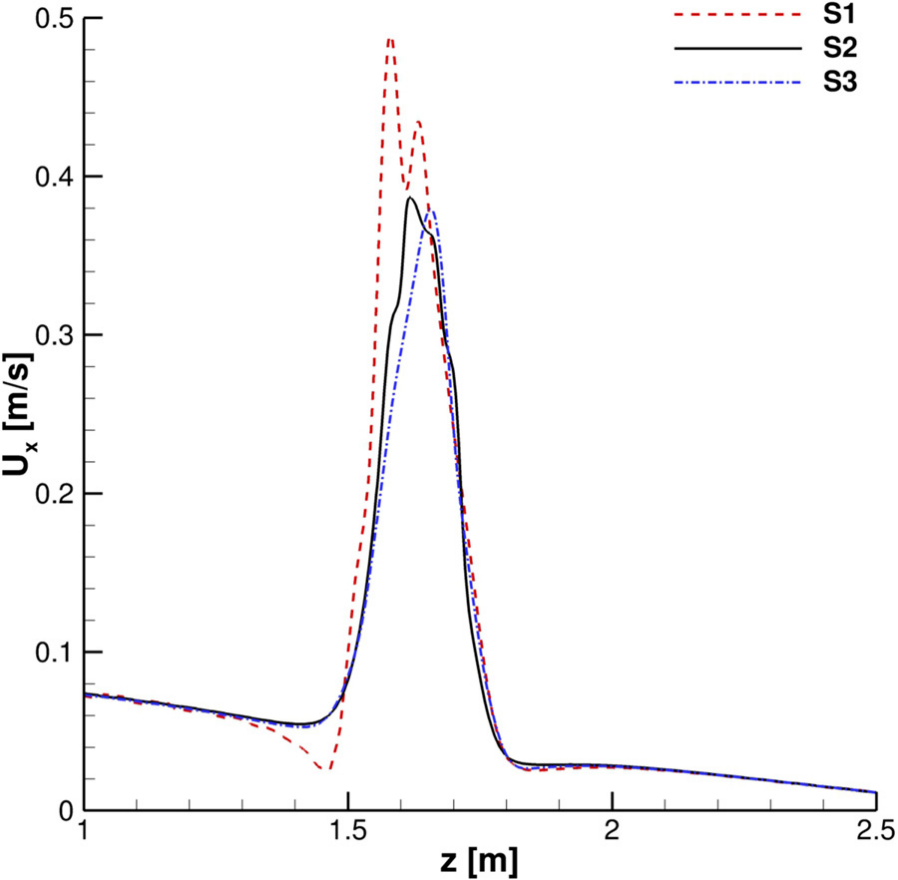
Streamwise velocity profiles. *x* = 0.5 m, *y* = 0 m, and *t* = 1 s.

**FIG. 7.**
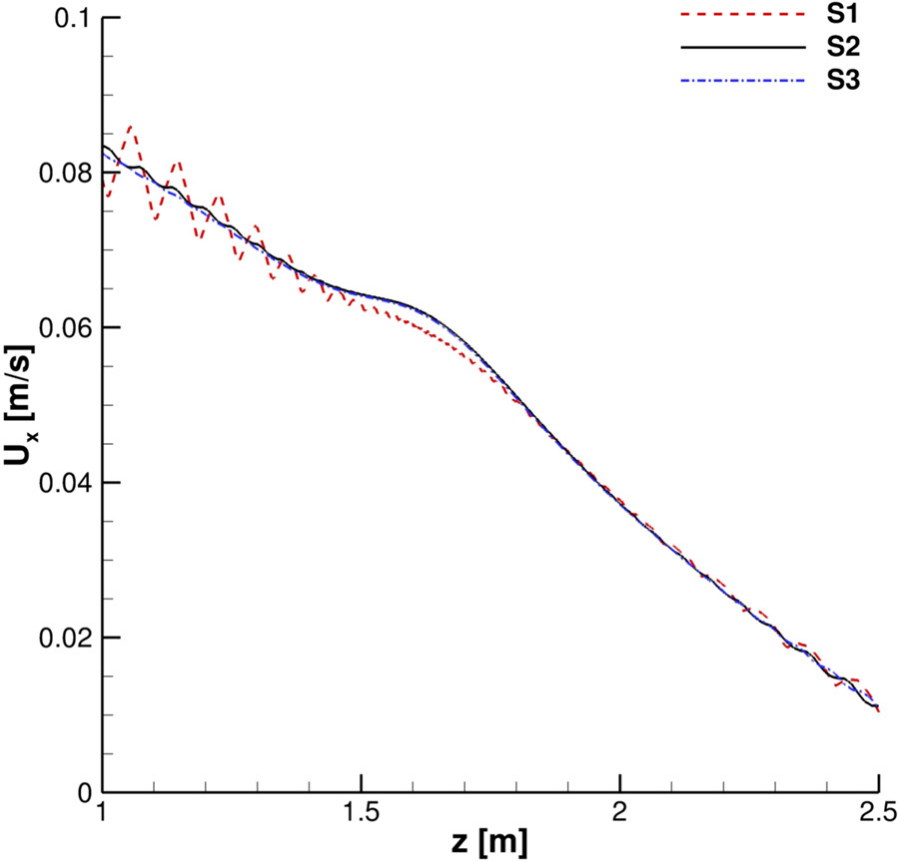
Streamwise velocity profiles. *x* = 1 m, *y* = 0 m, and *t* = 1 s.

A grid convergence study was carried out. Streamwise velocity profiles for the continuous phase were collected in order to evaluate the time evolution of the cough. In Figs. 4 and 5, the streamwise velocity, *U*_*x*_, behavior in the mouth print proximity is shown: *S*2 and *S*3 results are in good agreement both during the ejection phase and immediately after it has occurred. The *S*1 grid, instead, produces unphysical fluctuations in the entertainment zone. This is also true when the primary flux evolves into the domain (Figs. 6 and 7).

**FIG. 8.**
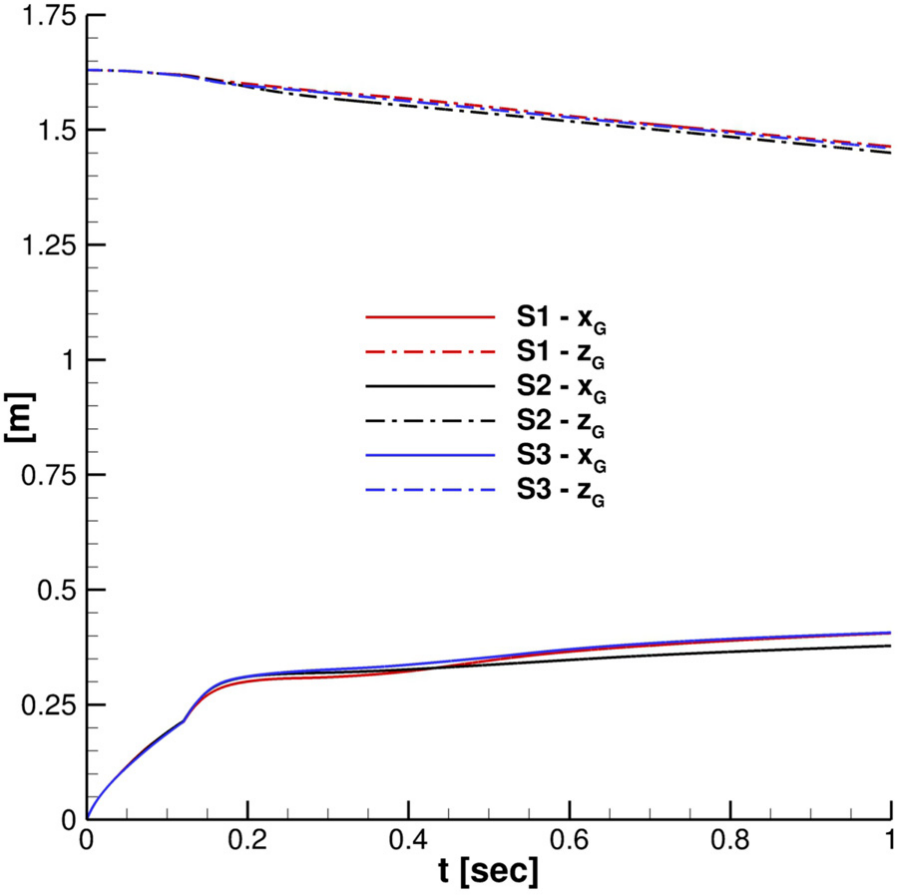
Grid effect. Particles' cloud properties: center of mass positions.

**FIG. 9.**
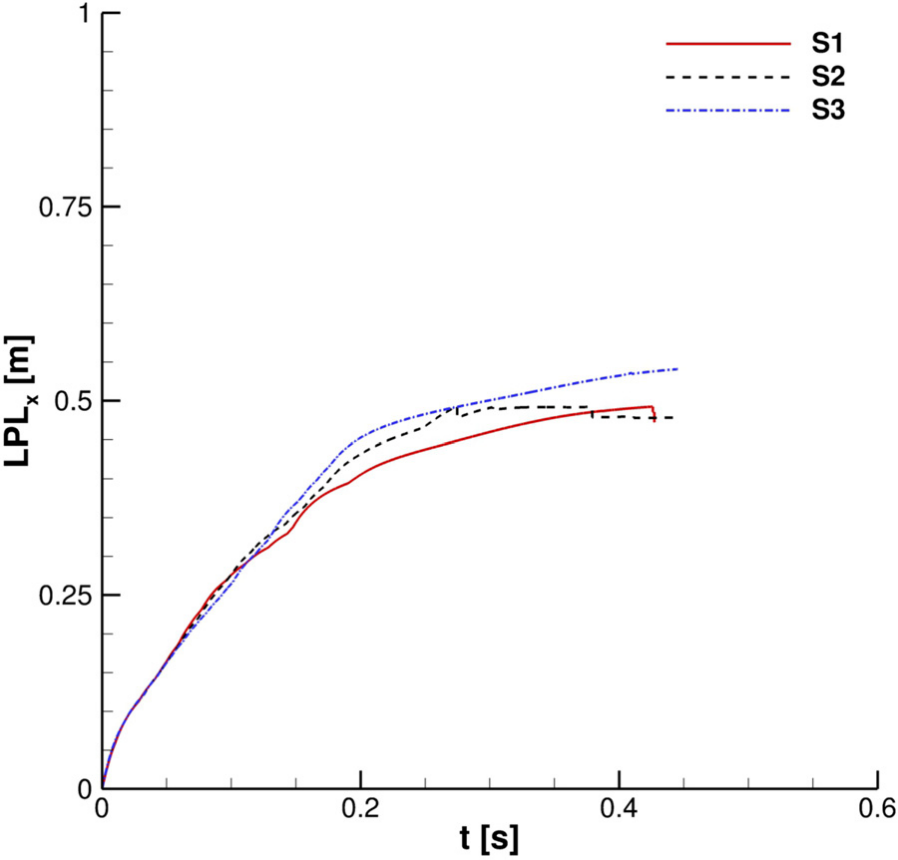
Grid effect. Particles' cloud properties: streamwise liquid penetration length.

For what concerns the cloud development, its center of mass position and LPL_x_ time evolution were monitored. Examining Fig. 8 is easy to see that *S*2 and *S*3 grids provide very similar behavior. This is almost confirmed from the comparison in Fig. 9. For this, *S*2 was chosen as the best compromise between solution accuracy and computational load for all the following simulations. It is important to point out that for the entire grid convergence study, Co_max_ = 0.04.

### B. Courant number effect

**FIG. 10.**
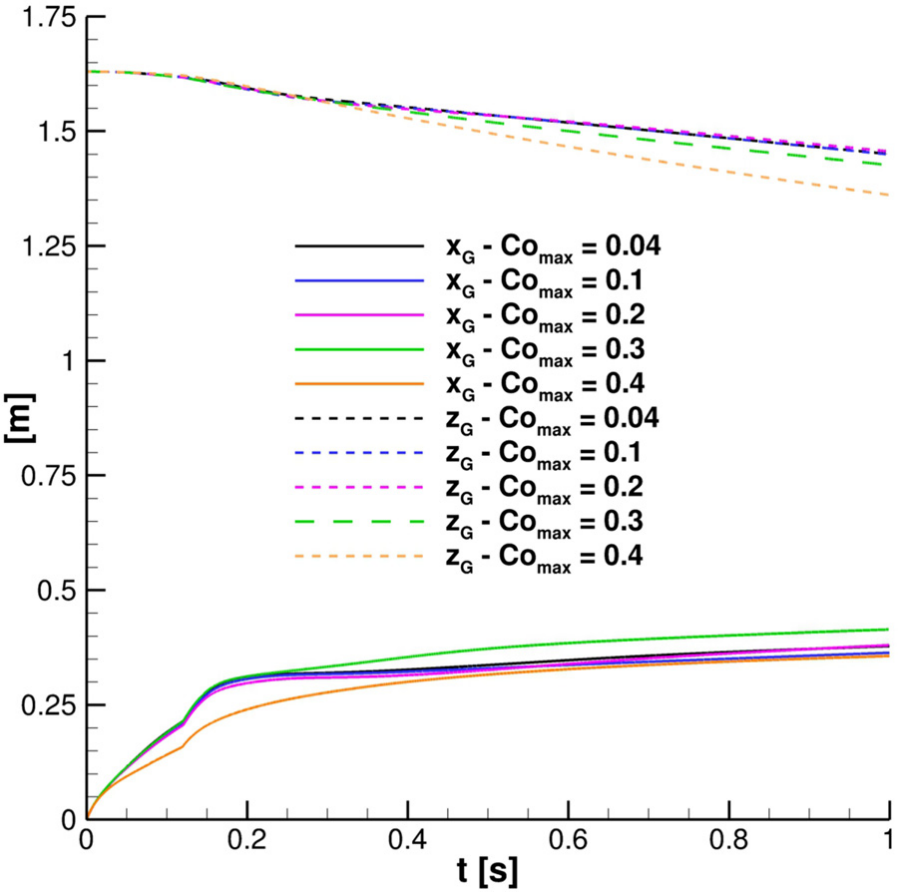
Co number effect. Particles' cloud properties: center of mass positions.

**FIG. 11.**
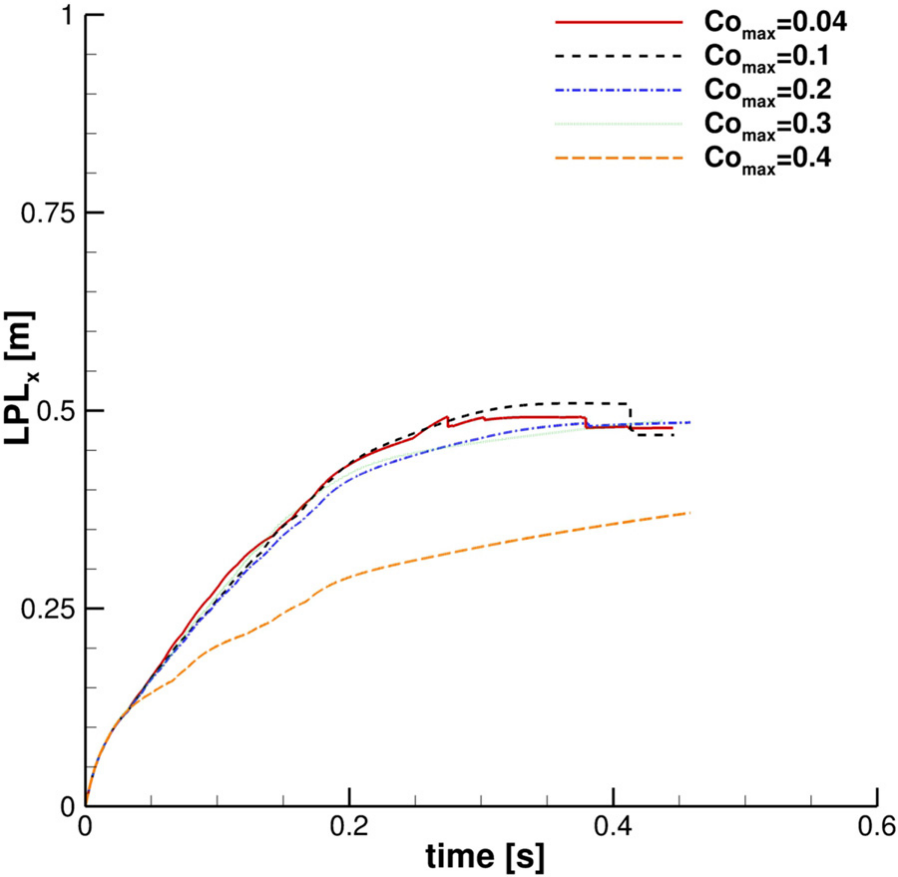
Co number effect. Particles' cloud properties: streamwise liquid penetration length.

In the context of the dynamic adjustable time stepping technique adopted in this work, the evaluation of the correct Co_max_ is crucial. Five different maximum Courant numbers: 0.04, 0.1, 0.2, 0.3, and 0.4 were evaluated, and their influence on the cloud properties was analyzed. Figure 10 puts in evidence that for Co_max_ ⩽ 0.2, **G** shows the same trajectory. In fact, *x*_*G*_ as well as *z*_*G*_ time evolution are almost indistinguishable for the first three cases. A slightly different trend was found in the streamwise liquid penetration length (Fig. 11). In this case, LPL_x_, provided by assuming Co_max_ = 0.2, does not perfectly replicate the behavior given using lower ones. However, considering that the LPL_x_ behavior is slightly affected and the overall good results, Co_max_ = 0.2 is chosen for the investigations, even for ensuring acceptable computing times.

### C. Particles per parcel effect

**FIG. 12.**
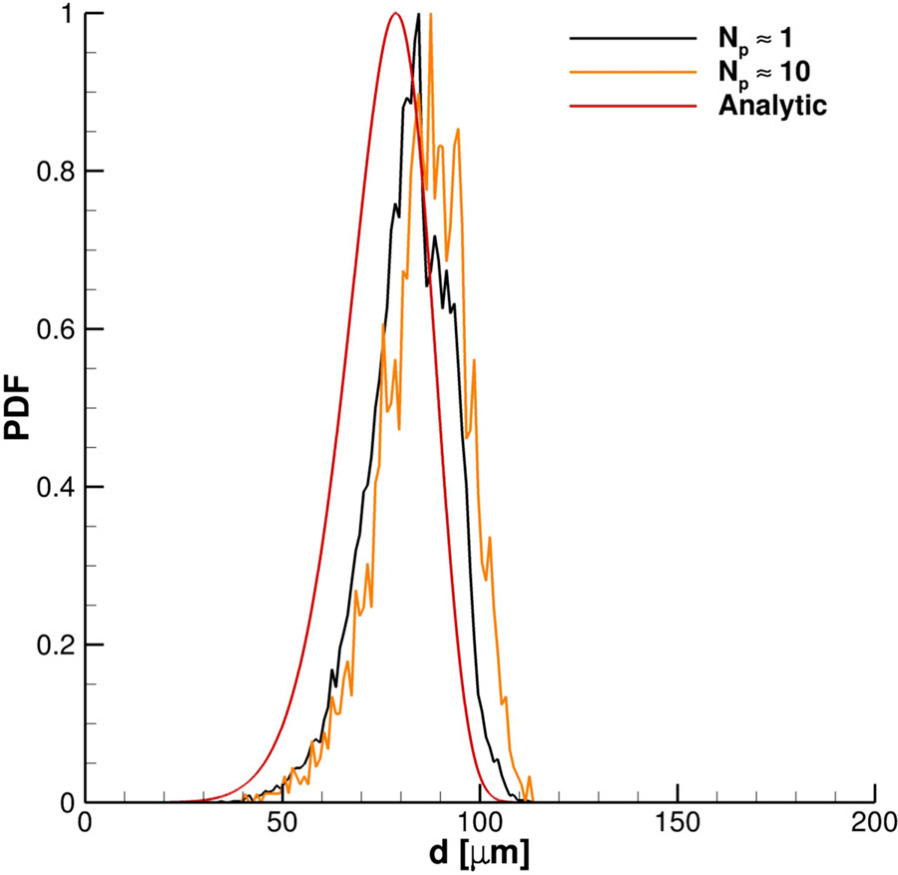
Effect of the number of particles per parcels on diameter PDF.

In the previously discussed analyses, a mean number of particles per parcel ${\bar{N}}_{p,i}\approx 10$ were considered for the sake of efficiency in the Eulerian–Lagrangian framework coupling. The intent is to complete the simulation parameter setting by means of the evaluation of the ${\bar{N}}_{p,i}$ effect on the droplets' cloud development. It is obvious that for ${\bar{N}}_{p,i}\to 1$, each particle is independent in its interaction with the carrier fluid, and, consequently, the cloud parameters are not affected by approximations due to the PSI–cell method parcel based implementation. Moreover, as noticeable in Fig. 12, using ${\bar{N}}_{p,i}\approx 1$, the diameters of the particles ejected during coughing better fit the analytic Rosin–Rammler probability density function (PDF) distribution.

Finally, ${\bar{N}}_{p,i}\approx 1$ is adopted for the purpose of achieving cloud proper modeling in terms of its dynamical behavior and PDF diameter size.

### D. Saliva droplet transport and interaction with UV-C light

**FIG. 13.**
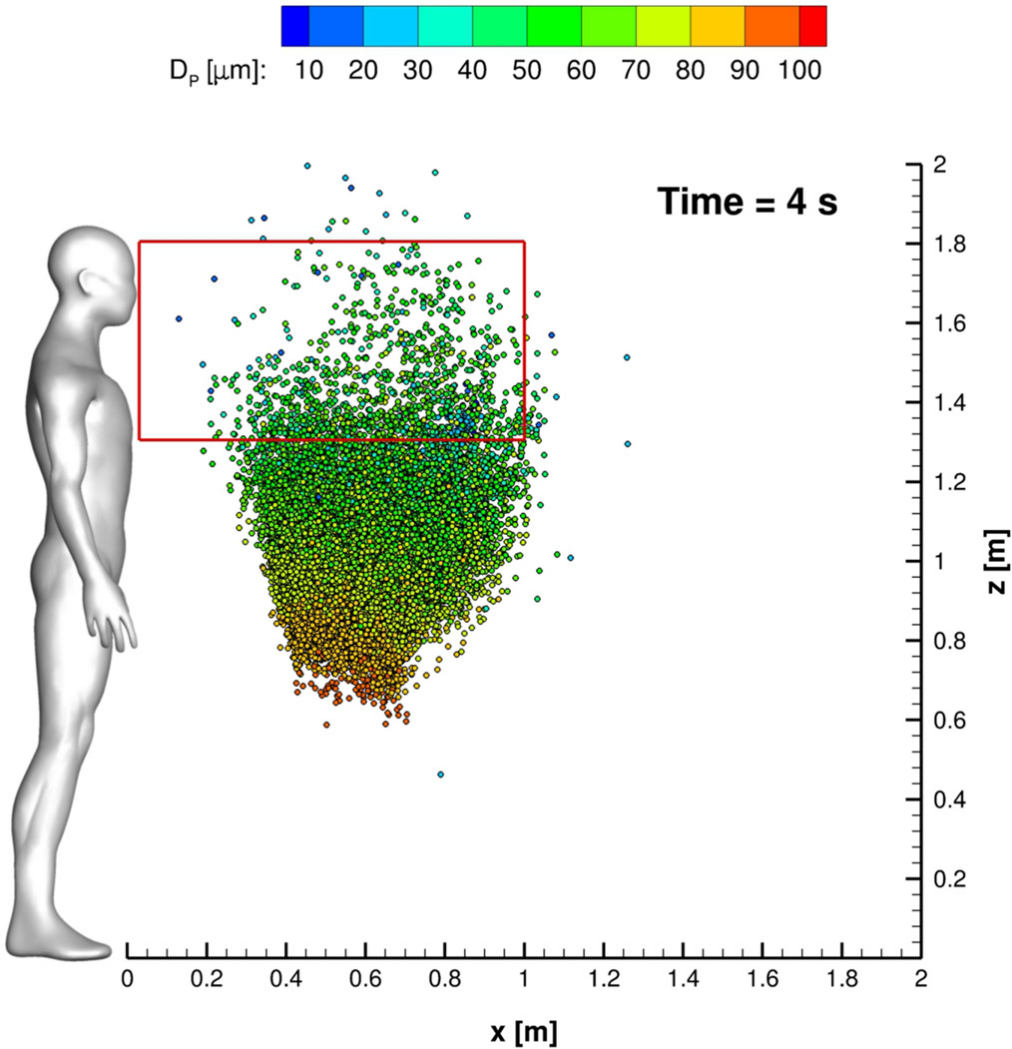
Cloud representation at *t* = 4 s. Parcels are colored with the particle diameter. The red rectangle is the Ω_2_ volume footprint.

**FIG. 14.**
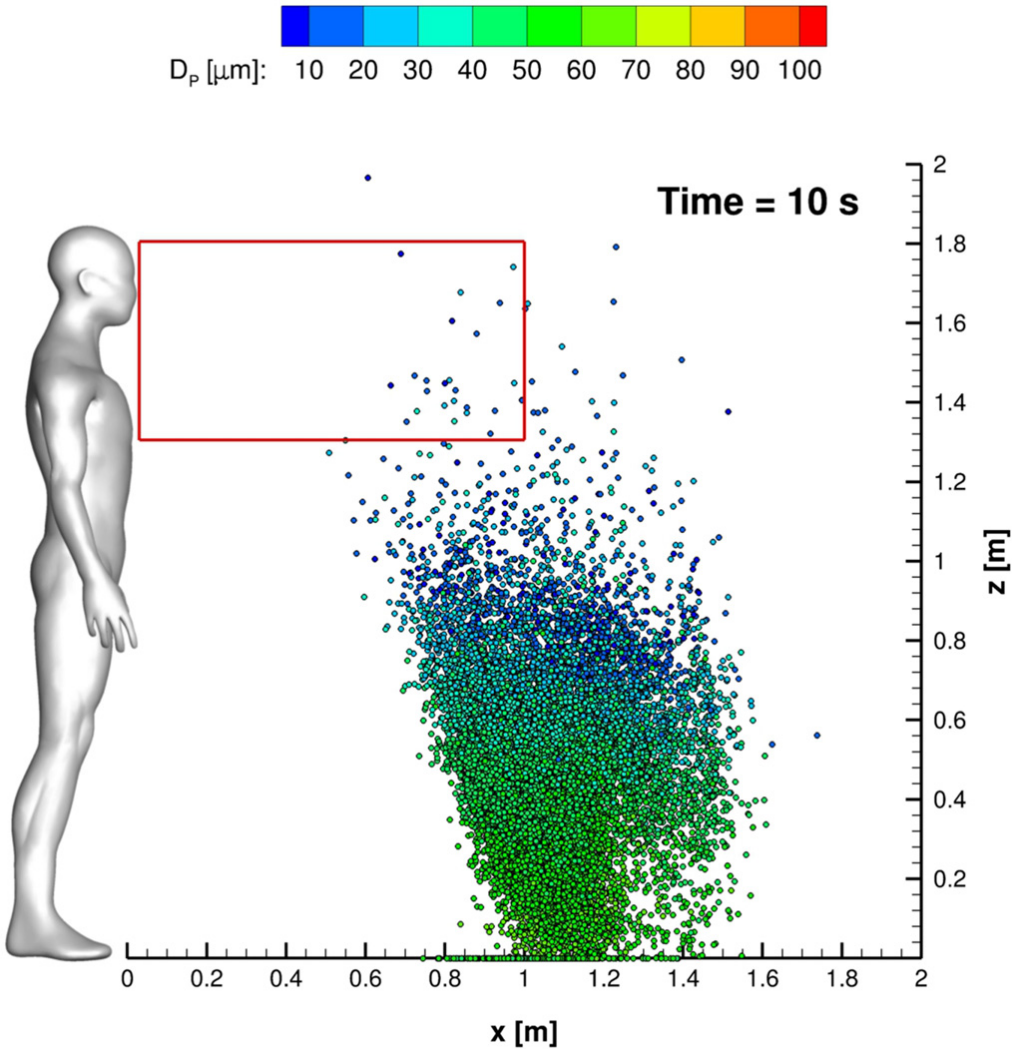
Cloud representation at *t* = 10 s. Parcels are colored with the particle diameter. The red rectangle is the Ω_2_ volume footprint.

**FIG. 15.**
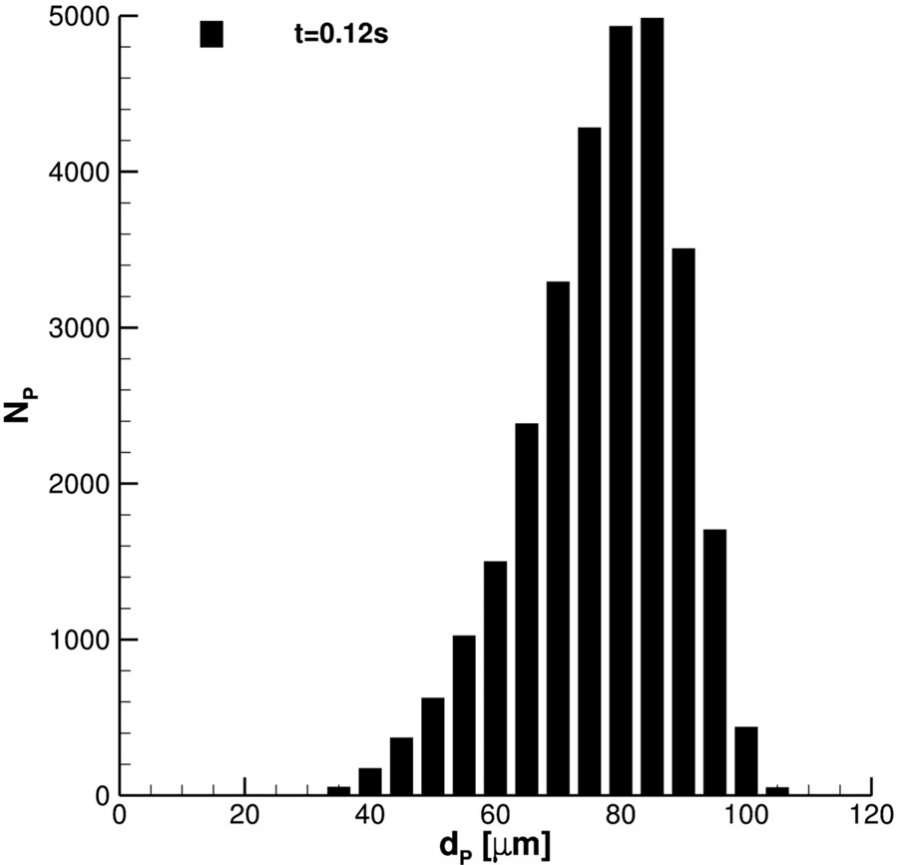
Particles' diameter distribution at *t* = 0.12 s. Single cough ejection. *D*_*min*_ = 33.47*µ*m, *D*_*max*_ = 108.90*µ*m, and ${\bar{D}}_{p}=81.37\;\mu m$.

**FIG. 16.**
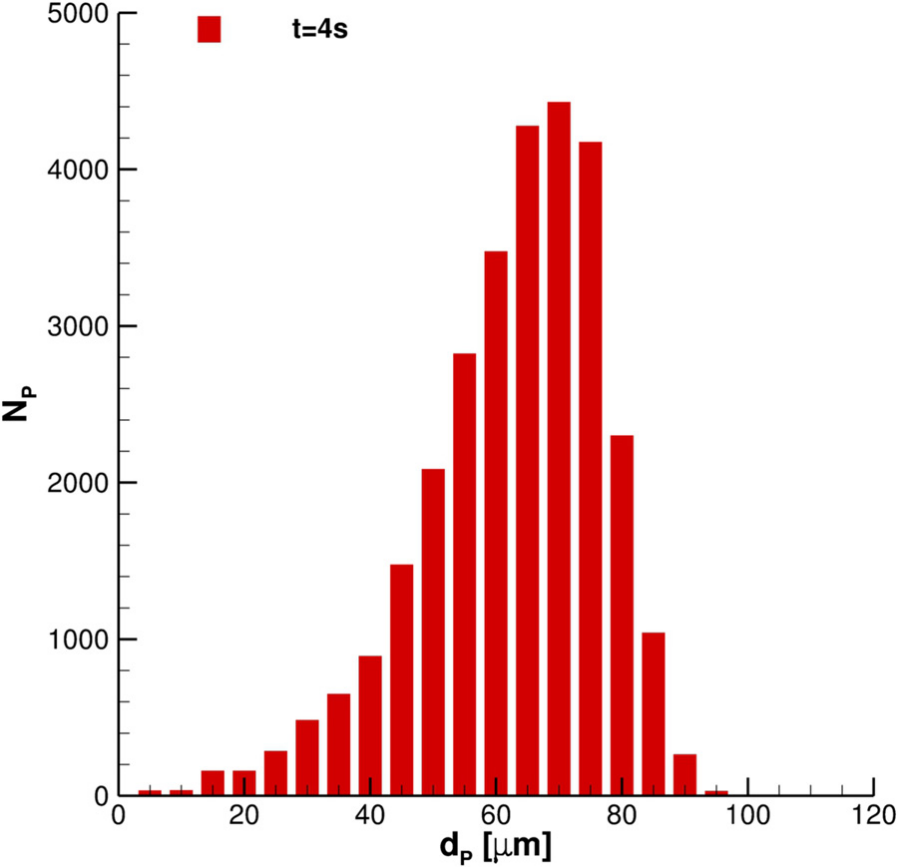
Particles' diameter distribution at *t* = 4 s. Single cough ejection. *D*_*min*_ = 2.85 μm, *D*_*max*_ = 98.34 μm, and ${\bar{D}}_{p}=67.81\;\mu m$.

**FIG. 17.**
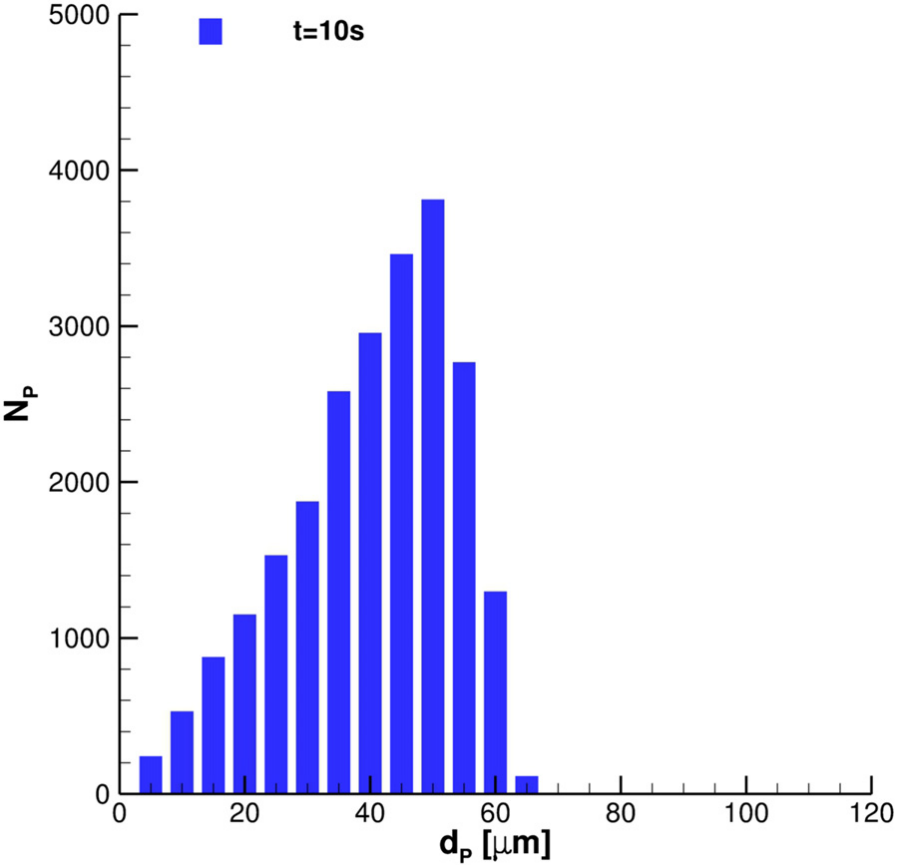
Particles' diameter distribution at *t* = 10 s. Single cough ejection. *D*_*min*_ = 1.06 μm, *D*_*max*_ = 66.45 μm, and ${\bar{D}}_{p}=43.07\;\mu m$.

In this subsection, all relevant features, concerning SARS-CoV-2 transmission of saliva cloud derived from coughing, are examined. A representation of the computed cloud for one cough ejection is shown in Figs. 13 and 14. It is very clear that the particles' diameter varies in time due to evaporation. This effect is analyzed quantitatively in Figs. 15–17, where bar charts of particles' occurrence, inside diameter bins with an amplitude of 5*µ*m, are depicted. At *t* = 0.12 s, the number of parcels having a diameter lower than 40*µ*m is almost negligible. The scenario changes radically over time. Figures 16 and 17 show that particles' diameter distribution moves toward smaller bins; at *t* = 10 s, also 0 ≤ *D*_*P*,*i*_ ≤ 5*µ*m bin is significantly populated.

Note that the analyses will not be limited to a single cough ejection but also to multiple ones. Up to three cough cycles delayed by 0.38 s, one over the other^14^ are applied. All the presented results are obtained from numerical simulations based on the *S*2 grid and Co_max_ = 0.2, as shown in Secs. IV A and IV B. The simulated physical time, excluding the precursor configuration, is ∼18 s (depending on breathing) due to the fact that the run is stopped when no parcels are still in the domain. The total computation time of a single case is about 20 h run in parallel using 384 CPU cores on CRESCO6.

**FIG. 18.**
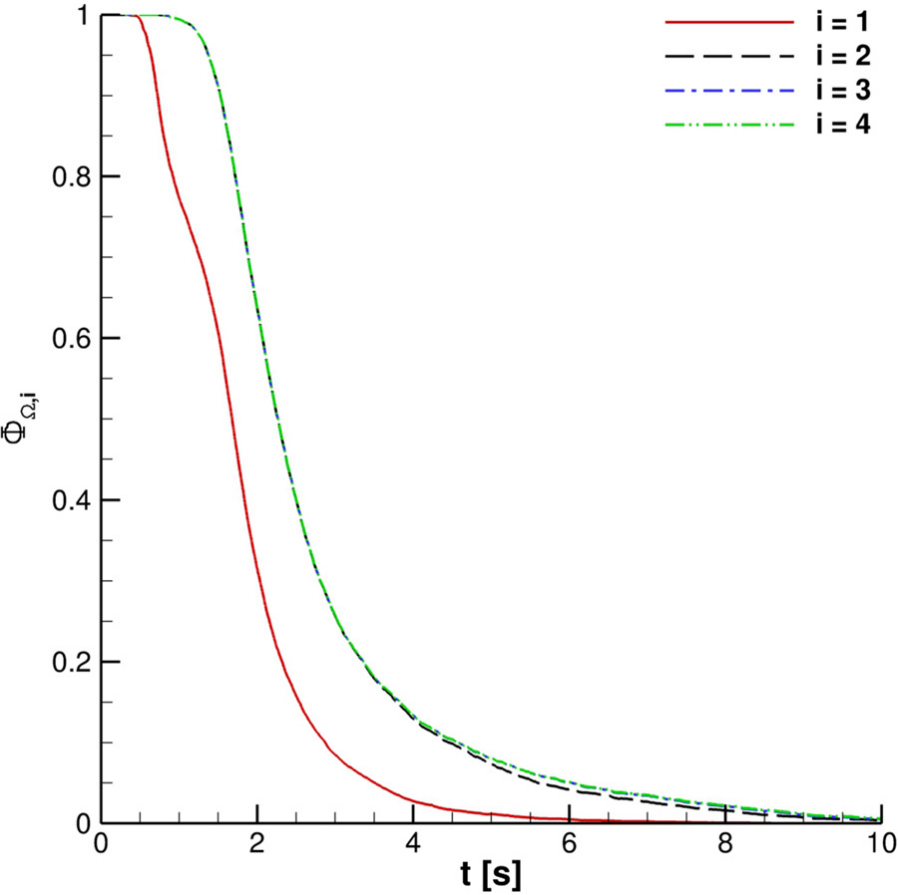
$\Phi_{\Omega_{i}}$ for one cough ejection.

**FIG. 19.**
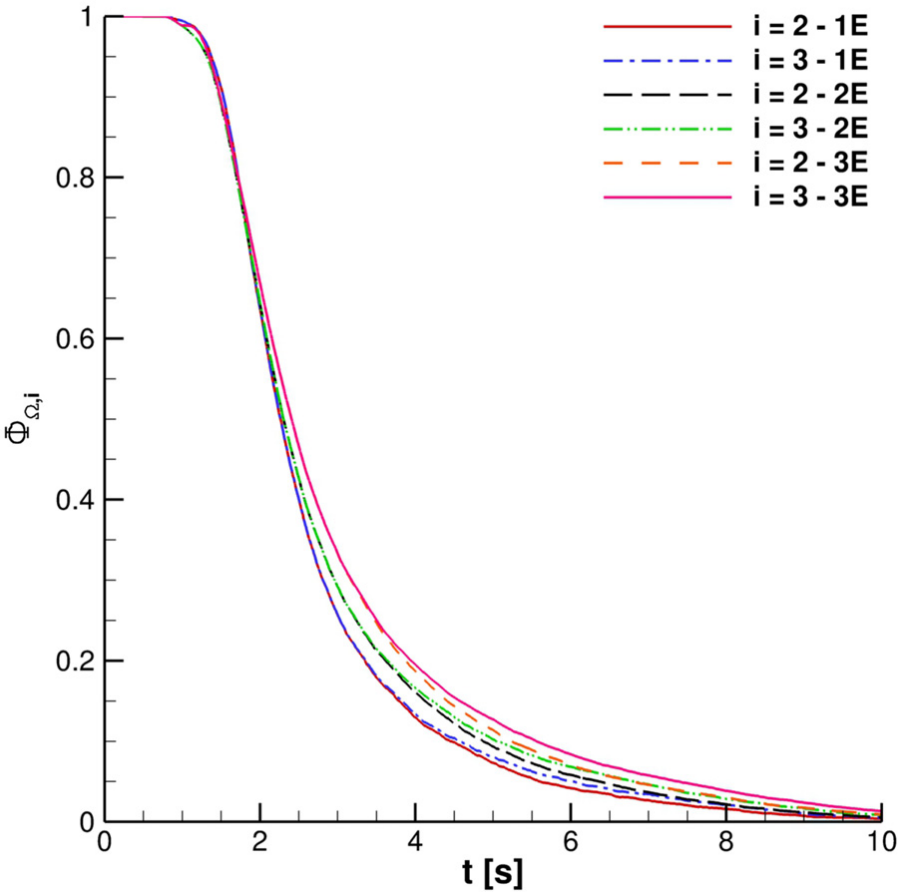
$\Phi_{\Omega_{i}}$ for multiple cough ejection. In the legend, 1*E* is for one ejection, 2*E* is for two ejections, and 3*E* is for three ejections.

Figure 18 depicts the fraction of particles present in the four reference volumes defined at the beginning of this section. It is really interesting to note that $\Phi_{\Omega_{i}}$ curves are very close for 2 ≤ *i* ≤ 4. Therefore, regarding the possibility to receive infected particles when the external wind is not present, distances ranging from 1.0 m to 1.5 m are equivalent. A similar condition is highlighted in the case of multiple cough ejections. Figure 19 shows that similar droplets' contamination is produced for Ω_2_ and Ω_3_ for different coughing; Ω_4_ results are not included in the plot to avoid the unreadability of the chart.

**FIG. 20.**
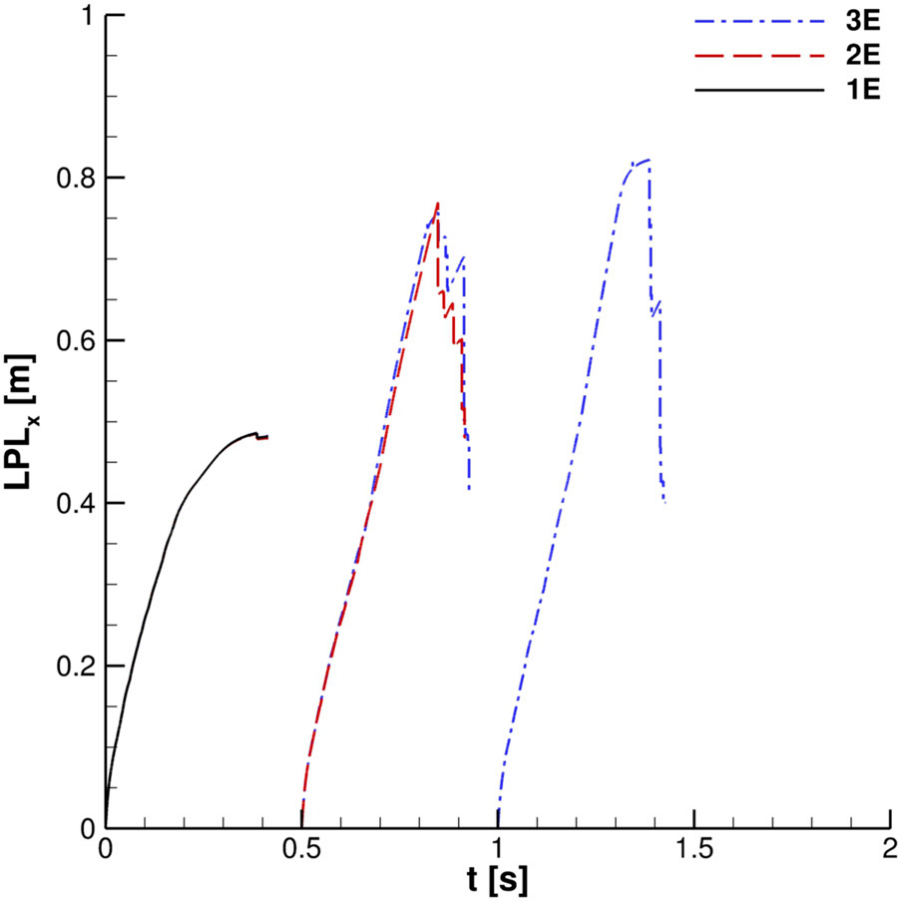
Streamwise liquid penetration length. For legend details, see Fig. 19 caption.

In Fig. 20, streamwise liquid penetration length, LPL_x_, is depicted. In the case of single saliva droplets' ejection, $O\left({LPL}_{x}\right)≃0.5\;m$ is found; differently, when the injections' number increases, $O\left({LPL}_{x}\right)$ rises to around 0.8 m. It is also interesting to note that, after the first cough, the LPL_x_ curve slope grows significantly and the largest emitted particles travel not more than 1 m.

**FIG. 21.**
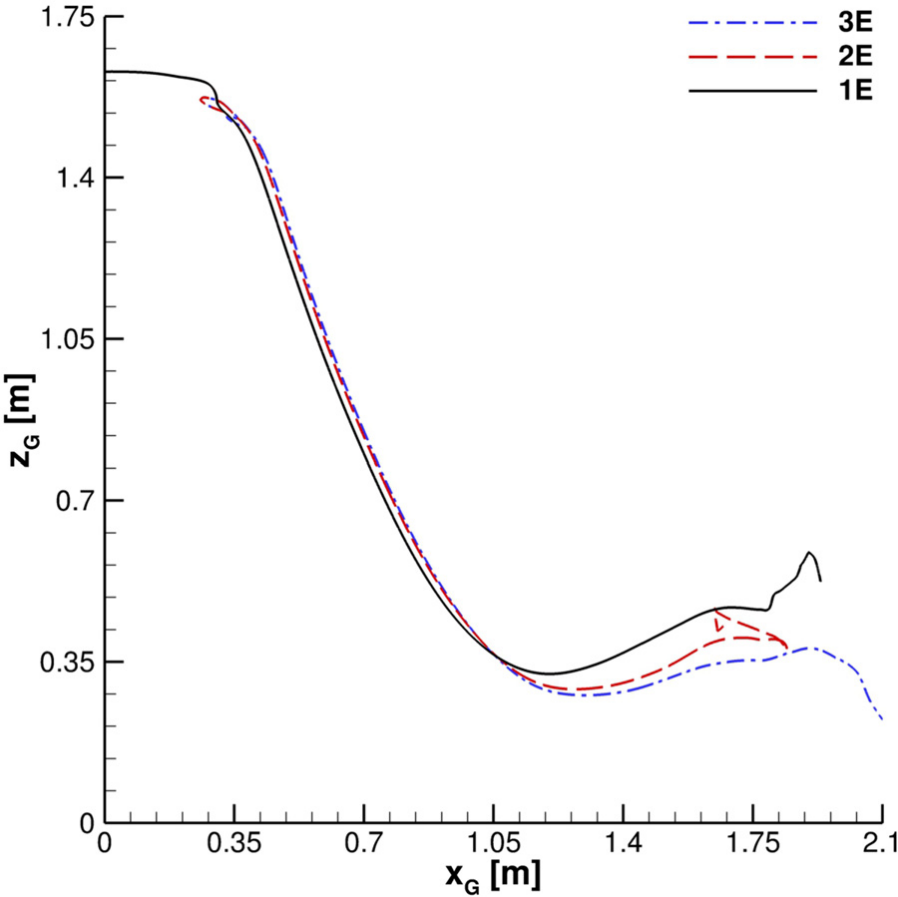
Cloud center of mass trajectory. For legend details, see Fig. 19 caption.

**FIG. 22.**
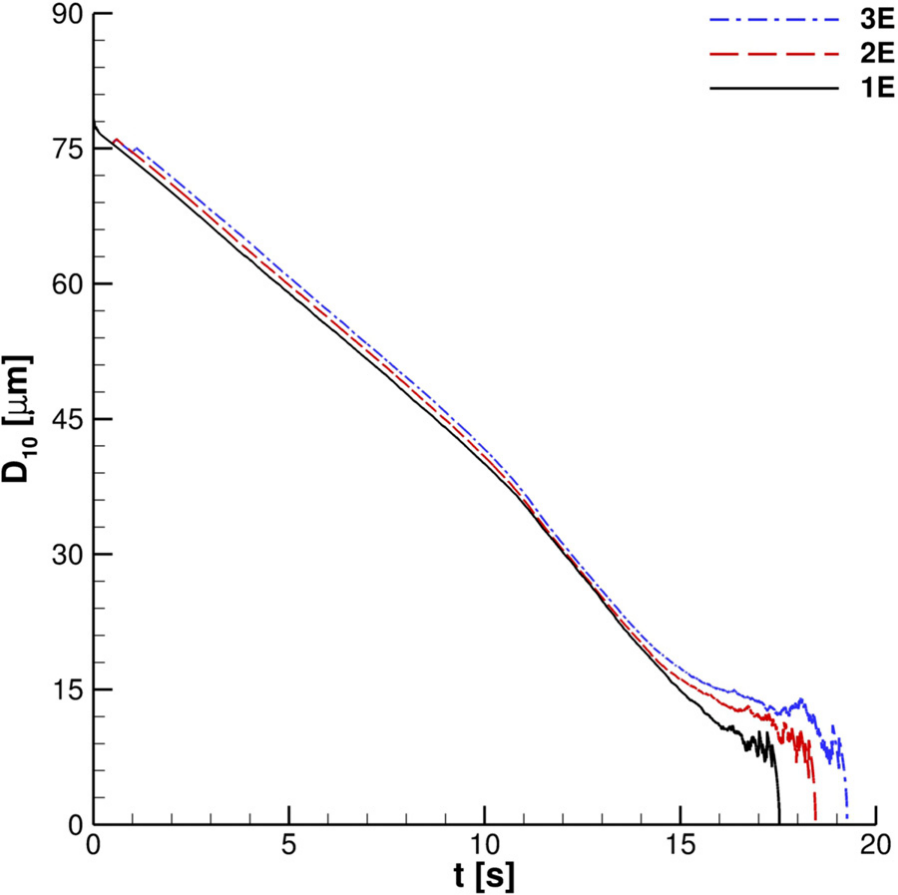
*D*_10_ time history. For legend details, see Fig. 19 caption.

The center of mass trajectory on the *x*–*z* plane is shown in Fig. 21. The number of cough ejections does not affect *x*_*G*_, *z*_*G*_ evolution largely, except for its last portion. For *x*_*G*_ ≃ 0.35 m, the *x*_*G*_, *z*_*G*_ curve underlines a clear trend change. Actually, in the proximity of this critical point, the trajectory changes from an almost linear up to *x*_*G*_ ≃ 1 m. This is due to the cancellation of the inertial term. In the last part of the curve, the evaporation and parcels' interaction with the bottom wall lead to a complex behavior. The center of mass trajectory suggests that several particles are located at distance over 1.5 m from the emitter. Nevertheless, a very limited number is situated within the range 1.3 m ≤ *z* ≤ 1.8 m. In *z* ≤ 1.3 m, several droplets, characterized by gradually decreasing diameters, can be found. This phenomenon is analyzed through *D*_10_ time evolution for different cough ejections (see Fig. 22). In our computations, *D*_10_ approaches average particles' diameters since we use ${\bar{N}}_{p,i}\approx 1$. Consequently, for *t* > 15 s, the parcels laden into the domain reach very small diameters and, a few seconds later, completely evaporate. This behavior corroborates that particles having very small diameters, which are reached into the domain after evaporation, remain in suspension for a limited time in accordance with Busco *et al.*^16^

**FIG. 23.**
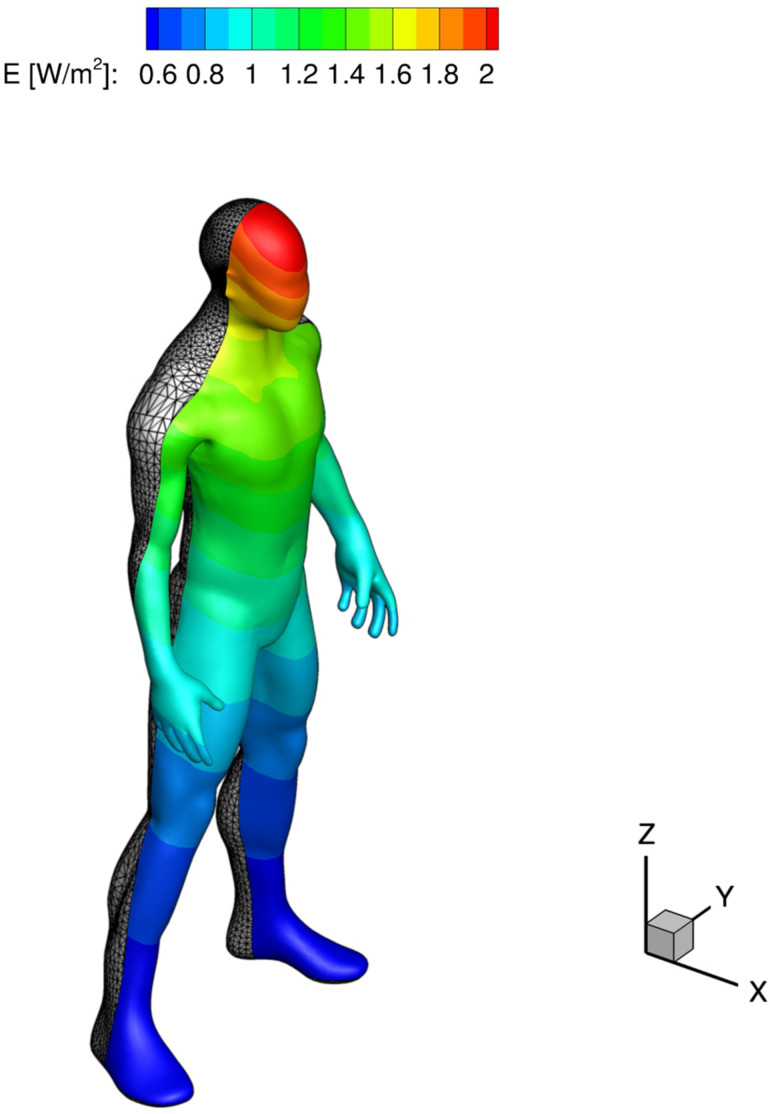
UV-C intensity field received by mannequin placed at the domain inlet region. Crossflow oriented 55 W lamp.

Another aim of this work is to evaluate the consistency between UV-C viral inactivation efficiency and droplets' residence time in a reference volume. The biological inactivation produced by UV-C light is analyzed by considering all the saliva droplets introduced in the domain as fully active. A commercial cylindrical lamp (having a radius of 1.4 cm and a length of 90 cm) is used as the UV-C source. It is positioned at the domain top, and different lamp layouts were investigated. In the first case, the streamwise orientation was fixed and the closer lamp edge to the mouth print is positioned in the point $\left(0.5\;m,0\;m,3\;m\right)$. In the second configuration, the lamp is aligned to the crossflow direction and its center is placed at $\left(1\;m,0\;m,3\;m\right)$. Also, two different lamp powers, *W*_*l*_, were considered: 25 W and 55 W. These values were selected in order to give a not risky UV-C dose to a possible cough emitter in accordance with the Ultraviolet Radiation Guide published by Navy Environmental Health Center (USA).^39^ Looking at Table II, it is possible to observe the average and maximum UV-C dose (received in 10 s) related to mannequin placed at the domain inlet region. Table entries, connected to the average values, are deduced from a numerical integration of UV-C field irradiating the space discretized mannequin (only in its anterior portion) represented in Fig. 23.

**TABLE II.** Average and maximum UV-C dose (in 10 s) for the mannequin represented in Fig. 23.

Case	Avg. dose (J/m^2^)	Max. dose (J/m^2^)
Streamwise–55W	8.37	15.21
Crossflow–55W	10.16	20.89
Crossflow–25W	4.62	9.49

**FIG. 24.**
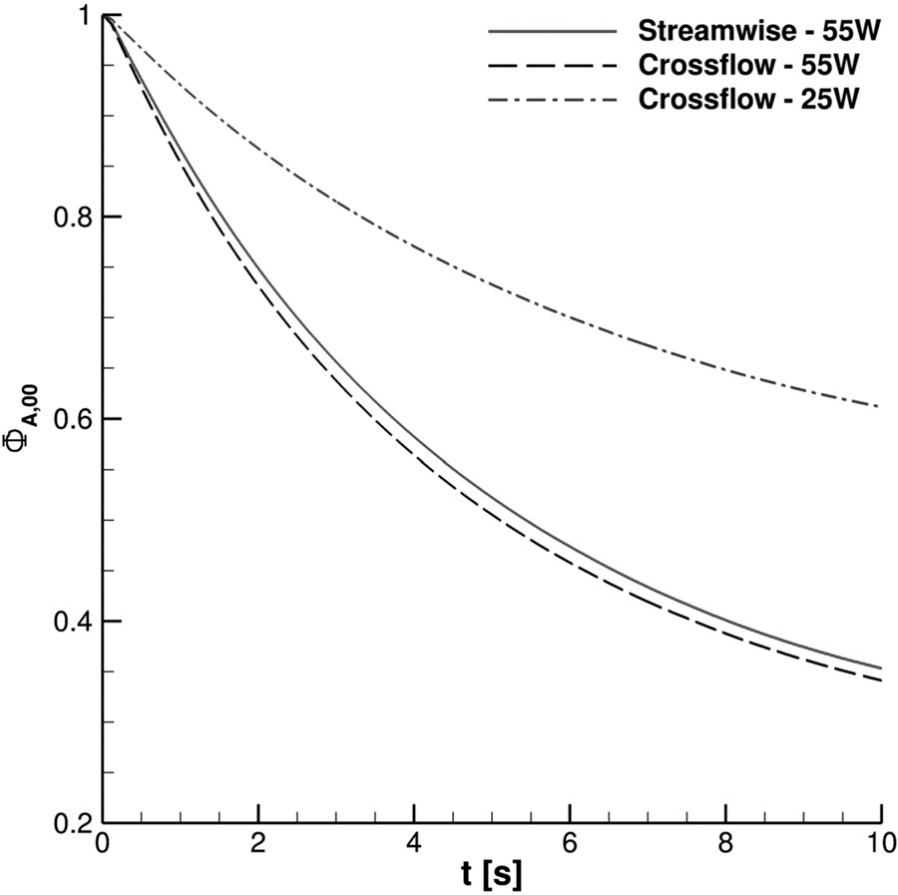
Effect of UV-C lamp power.

**FIG. 25.**
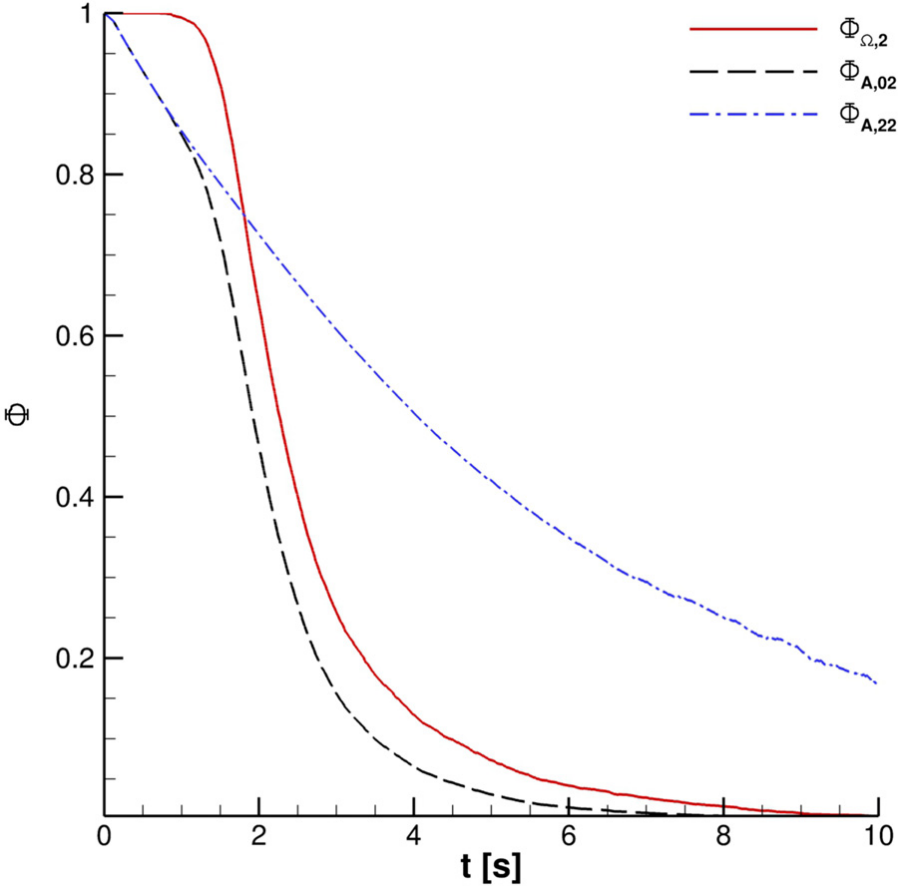
Effect of UV-C radiation for one cough ejection.

**FIG. 26.**
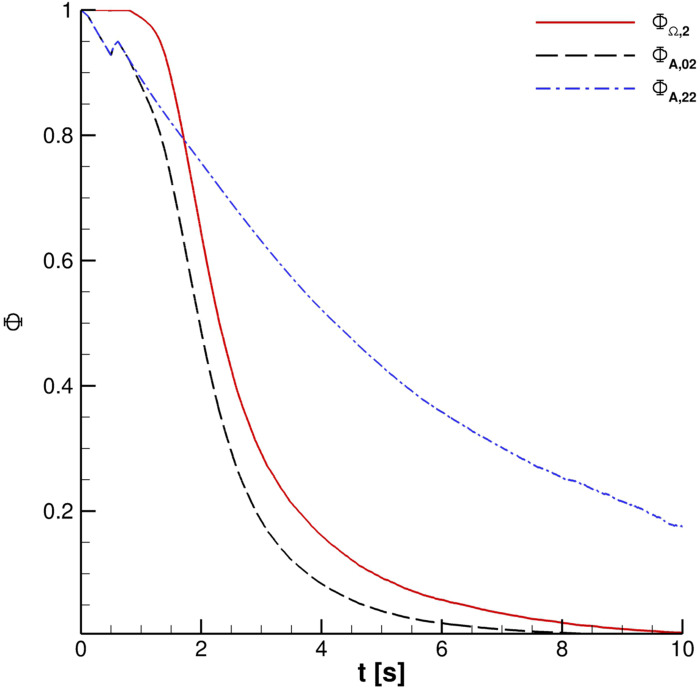
Effect of UV-C radiation for two cough ejections.

**FIG. 27.**
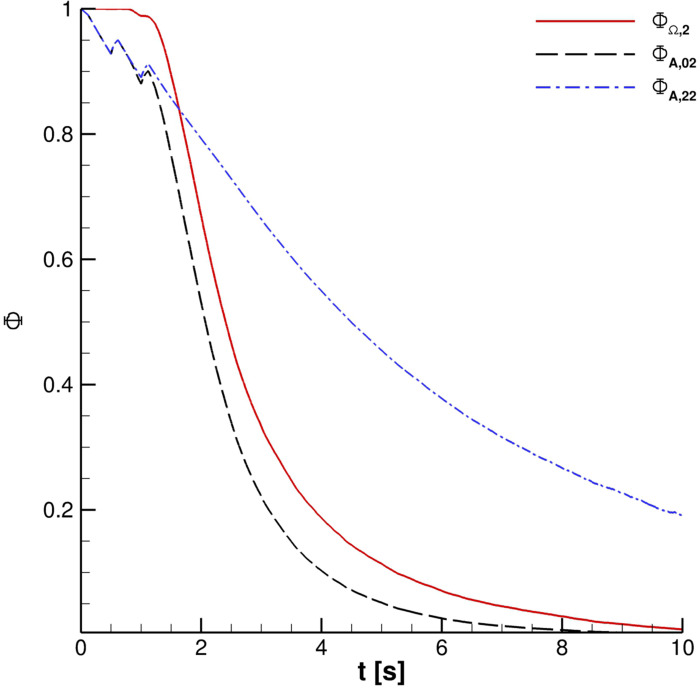
Effect of UV-C radiation for three cough ejections.

The effect of the UV-C lamp and orientation is clearly underlined in Fig. 24. The Φ_*A*,00_ behavior *vis-à-vis* time is evident for the reported cases. Anyway, the impact of the lamp orientation is barely noticeable; the lower power level lamp produces, according to the authors' opinion, a modest biological inactivation of parcels. Thus, hereinafter, the reference configuration uses a crossflow 55 W lamp. The UV-C radiation inactivation capabilities are shown in Figs. 25–27. Only Ω_2_ volume is studied since it is almost equivalent to Ω_3_ and Ω_4_ with regard to the droplets' presence. It is very important to stress that the permanence time of droplets has 10 s scale for different coughing. This condition can be also noted by observing Fig. 14. Furthermore, UV-C light has a very good impact on the reduction of active particles. Indeed, the Φ_*A*,22_ index rapidly decreases in time, reducing the contamination risk. In this context, it is interesting to note that after 4 s, the number of parcels in Ω_2_ is low and they are mainly located in its bottom side where UV-C field intensity suddenly decays, see Fig. 13. Therefore, the overall effect of this technique can be considered very promising.

## V. CONCLUSIONS

This paper addresses the development and application of an Eulerian–Lagrangian model for saliva droplets' cloud deriving from coughing in relation to SARS-CoV-2 transmission. An extensive convergence study in terms of grid spacing, time discretization, and main cloud parameters was performed in order to provide reliable results. This work focuses on no-wind configurations, thus a field initialization strategy was developed. This element is strongly needed to appropriately reproduce hydrostatic pressure and turbulence variables consistent with atmospheric conditions. It is worth noting that the cloud evolution is significantly influenced by the fields' initialization. In particular, uniform initial fields produce an underestimation of the cloud axial penetration.

In addition, droplet evaporation was examined by observing that particles reduce their diameter significantly in time until complete evaporation. Moreover, smaller droplet suspension does not occur for a very long time since they reach the floor in ∼17 s–20 s. In this regard, we have to note that smaller particles (*D*_*p*,*i*_ < 10 *µ*m) are not directly injected into the domain. Differently, they are generated due to evaporation in a zone closer to the floor. Thus, aerosols' settling time provided by the Stokes equation is not compatible with the problem studied here. The cloud kinematics was studied, after one or multiple cough ejections, in order to evaluate safety distances. The center of mass evolution and axial liquid penetration length were computed. It was highlighted that several particles are located at distances over 1.5 m from the emitter. Nevertheless, only few particles are positioned in the vertical range 1.3 m ≤ *z* ≤ 1.8 m already after 4 s. Therefore, two new indices were introduced in order to provide quantitative information about the contamination risk. In light of the above-mentioned data, we noted that only a few particles get to distances greater than 1.0 m, in the vertical range 1.3 m ≤ *z* ≤ 1.8 m, after different breathing conditions. Hence, it may be confirmed that social distances greater than 1.0 m can suffice to reduce, notably, the possibility of receiving infected particles under not windy conditions.

Finally, the biological inactivation, produced by UV-C radiation at 254 nm, was investigated. A new approach to model the UV-C inactivation effect in an Eulerian–Lagrangian framework was presented. The possibility to efficiently inactivate SARS-CoV-2, in a time-scale consistent with the droplets' residence time in a specific volume, was demonstrated. A safe UV-C dose was provided; thus, this technique is a promising approach to perform a real-time disinfection of a cloud resulting by coughing.

## Data Availability

The data that support the findings of this study are available from the corresponding author upon reasonable request.
